# Analysis and Characterization of Kisspeptin and Its Analogues in Serum and Urine Samples by Liquid Chromatography–High‐Resolution Mass Spectrometry for Doping Control Purposes

**DOI:** 10.1002/dta.70081

**Published:** 2026-04-29

**Authors:** Sophia Krombholz, Linus Korsmeier, Andreas Thomas, Mario Thevis

**Affiliations:** ^1^ Institute of Biochemistry, Center for Preventive Doping Research German Sport University Cologne Germany; ^2^ European Center for Emerging Doping Agents Cologne Germany

**Keywords:** anti‐doping, kisspeptin, LC‐HRMS, peptide analysis

## Abstract

The use of testosterone‐stimulating peptides for doping purposes is prohibited for male athletes by the World Anti‐Doping Agency (WADA). Among these substances is kisspeptin (KP‐54), its isoforms (KP‐14, KP‐13, and KP‐10), and synthetic receptor agonists such as TAK‐448. Thus, they have been included in the WADA Prohibited List in 2024. To enable effective detection of kisspeptin misuse, reliable analytical methods are required. Consequently, this study aimed to develop liquid chromatography–high‐resolution mass spectrometry‐based (LC‐MS) methods for the detection of kisspeptin and its analogues in human serum and urine. In addition, peptide stability in different biological matrices was investigated, and metabolic stability was characterized in vitro. Extraction methods based on cation‐exchange solid‐phase extraction were optimized, enabling a selective and sensitive LC‐MS analysis with limits of identification (LOI) ranging from 0.8 ng/mL (KP‐54) to 10 pg/mL (TAK‐448). Analysis of a reference population comprising *n* = 20 serum samples and *n* = 100 urine samples revealed no detectable signals of endogenous kisspeptins and/or its degradation products. All native kisspeptins investigated showed poor stability in urine and blood; however, several degradation products could be identified. These metabolites could serve as complementary target analytes and improve the detectability of kisspeptins in doping control samples. Overall, the study provides an important foundation for the confirmatory analysis of kisspeptins in doping controls and delivers in vitro insights into their metabolic behavior. Analysis of samples collected after administration of the peptides remains necessary to further assess the detectability and metabolic profiles in authentic samples.

## Graphical abstract

Sensitive LC‐HRMS methods were established for the detection of kisspeptin and its analogues in serum and urine, alongside their analytical and metabolic characterization. Stability studies were conducted in serum, plasma, and urine, highlighting the importance of appropriate sample handling for reliable anti‐doping analysis.

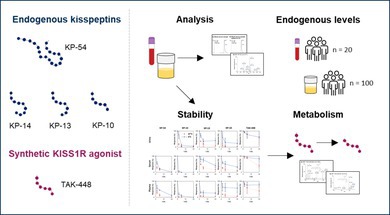

## 1 Introduction

**FIGURE 1:**
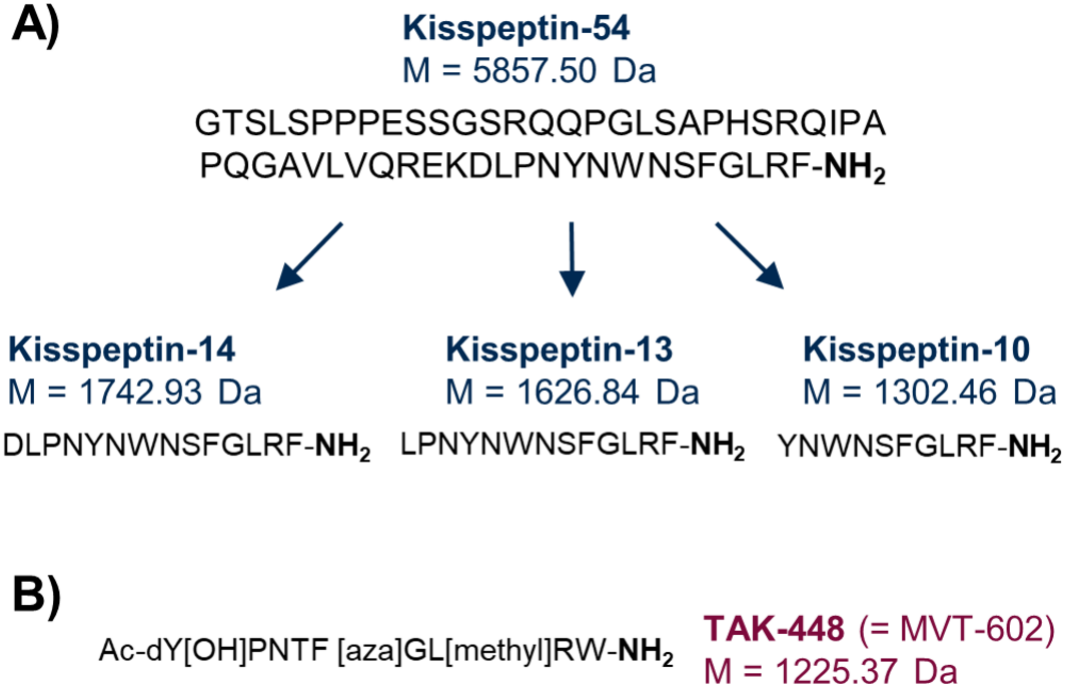
Primary sequences and average molecular masses of (A) the endogenous peptide hormones kisspeptin‐54, kisspeptin‐14, kisspeptin‐13, and kisspeptin‐10 and (B) the synthetic KISS1R agonist TAK‐448.

Kisspeptin is a neuropeptide and potent stimulator of the hypothalamic–pituitary–gonadal (HPG) axis [1]. By binding to the G‐protein‐coupled receptor KISS1R, it directly induces the release of gonadotropin‐releasing hormone (GnRH) into the human circulation, which in turn stimulates the release of luteinizing hormone (LH) and follicle‐stimulating hormone (FSH) in the anterior pituitary gland [2]. The peptide is encoded by the KiSS1 gene, which was originally identified as a suppressor of metastasis in malignant melanoma [3]. The discovery of the gene in Hershey (PA, USA) led to it being named after the famous chocolate “kisses” produced there, the “SS” additionally referring to the suppressor gene. Today, kisspeptin is known as a crucial regulator of puberty, the sex hormone‐mediated release of gonadotropins, and human fertility [1]. Consequently, substantial research has been directed towards the potential use of kisspeptin either for the treatment of hormone‐dependent disorders of sexual or reproductive functions (e.g., hypothalamic amenorrhea [HA], delayed puberty, and polycystic ovary syndrome [PCOS]) as well as hormone‐dependent cancers (e.g., prostate cancer and breast cancer) or as a biomarker for corresponding diseases [4]. Structurally, kisspeptin is composed of 54 amino acids with a C‐terminal Arg‐Phe‐NH_2_ motif, which is common to other neuropeptides [5]. In the circulation, kisspeptin (KP‐54) is further cleaved to peptides of 14 (KP‐14), 13 (KP‐13), and 10 amino acids (KP‐10), which all share the amidated C‐terminus [6]. Primary sequences of kisspeptin and its analogues are depicted in Figure 1. Although KP‐13 has only been investigated in preclinical testing, there are numerous clinical studies that have evaluated the therapeutic potential of KP‐54 and KP‐10 in reproductive disorders [7, 8, 9, 10, 11, 12]. The primary focus was their use in women with HA, PCOS, in vitro fertilization, or disorders of pregnancy. Within several investigations, it was shown that all native kisspeptins are inactivated relatively quickly by matrix metalloproteinases, which partially limits their therapeutic application [13, 14]. Therefore, an increasing number of synthetic KISS1R agonists are being developed and investigated with the aim of improved agonistic activity and metabolic stability through the modification of specific amino acids [14, 15, 16, 17]. One example is TAK‐683, which has a significantly higher receptor affinity than KP‐54 [18]. After it was discovered that the compound showed gel‐forming properties in aqueous solutions, it was further developed to TAK‐488, comprising several non‐natural amino acids substitutions including N‐terminal acetylation, D‐tyrosine, hydroxyproline, aza‐glycine, and methylated arginine (Figure 1) [16]. Consequently, the substance not only shows increased pharmacological activity but also significantly improved stability. Therefore, TAK‐448 (syn. MVT‐602) has already been studied in healthy women for its endocrinological effects [19]. However, the impact of kisspeptin and its analogues on the HPG axis is not only of clinical interest but also relevant in the context of anti‐doping. In males, the stimulation of LH‐dependent biosynthesis and secretion of testosterone results in supraphysiological levels of the androgen with potential effects on muscle mass and strength [20]. Consequently, kisspeptins may be misused for performance‐enhancing purposes by male athletes, and they were thus included in the Prohibited List of the World Anti‐Doping Agency (WADA) in 2024 [21]. This necessitates reliable analytical approaches; thus, the aim of this study was to develop sensitive methods for the detection of kisspeptin and its analogues in doping control samples via liquid chromatography–high‐accuracy/high‐resolution mass spectrometry. Based on their size, a detection of KP‐54 in human serum samples was intended, whereas for the smaller peptides (KP‐14, KP‐13, KP10, and TAK‐448), detection in human urine samples was evaluated. On the basis of literature indicating a limited stability of the peptides against serum proteases, a comprehensive investigation of their stability in human serum, plasma, and urine was undertaken. The aim was to identify degradation products of the peptides (i.e., metabolites) that could be suitable as (complementary) target analytes in doping controls in order to improve the detectability of the substances. Because in clinical studies kisspeptins were administered primarily by subcutaneous injection, metabolism in human skin S9 fraction was additionally studied. Although LC‐MS‐based methods for the determination of kisspeptins (e.g., KP‐10) have already been described [13, 22], endogenous kisspeptin concentrations have so far been assessed exclusively by immunological approaches [23, 24, 25]. Thus, a reference population comprising serum and urine samples from routine doping controls was analyzed in order to evaluate endogenous levels of kisspeptins using LC‐MS‐based analytical methods.

## 2 Material and Methods

### 2.1 Chemicals and Reagents

Reference material for human KP‐54 and TAK‐448 was purchased from Hycultec (Beutelsbach, Germany). Human KP‐54 was obtained from Biosynth (Bratislava, Slovakia), and KP‐13 and KP‐10 were from MedChem Express (Monmouth Junction, NJ, USA). As internal standards, rat amylin, purchased from TargetMol (Wellesley Hills, MA, USA) and d_4_‐Ala‐GHRP‐5, obtained from BMFZ (Düsseldorf, Germany) were used. Stock solutions (1 mg/mL) of all analytes were prepared in DMSO and stored at −20°C. For method development and validation, working solutions of 0.1 mg/mL were prepared in DMSO, aliquoted, and stored frozen at −80°C until usage. Human skin S9 fraction (total protein concentration: 5 mg/mL) was purchased from BioIVT (Westbury, NY, USA). Ammonia solution (25%) and dimethyl sulfoxide (DMSO) were from Carl Roth (Karlsruhe, Germany). Acetic acid, trifluoroacetic acid (TFA), and phosphate‐buffered saline (PBS) tablets were obtained from Sigma‐Aldrich (St. Louis, MO, USA). Acetonitrile (ACN), formic acid (FA), and methanol (MeOH) were purchased from VWR Chemicals (Radnor, PA, USA). Aqueous solutions were prepared using ultrapure water obtained from a Barnstead GenPure water purificator. For in vitro experiments and method validation, urine and serum samples were collected from healthy human volunteers. The sampling was approved by the ethics committee of the German Sport University Cologne (#139/2021), and all participants provided written consent.

### 2.2 Extraction of Kisspeptin‐54 From Serum Samples

KP‐54 was extracted from serum by means of mixed‐mode weak cation‐exchange (HR‐XCW) solid‐phase extraction (SPE). A 200 μL aliquot of serum was fortified with 10 μL of rat amylin (ISTD 1) solution to a final concentration of 50 ng/mL. For protein precipitation, 400 μL of a mixture of ice‐cold ACN/MeOH (1:1, v/v) was added, followed by thorough vortexing and centrifugation at 16,000 × *g* for 10 min. For SPE, Chromabond HR‐XCW columns (1 mL/30 mg, 45 μm) from Macherey‐Nagel (Düren, Germany) were pre‐conditioned with 1 mL of MeOH and 1 mL of water. Subsequently, the supernatant of the sample was added, diluted with 600 μL of water, and slowly drawn through the column. After washing with 1 mL of water and 1 mL of MeOH, elution was performed with 1 mL of 0.5% TFA in MeOH/water (80:20, v/v). After neutralization of the eluate with 4 μL of NH_3_ solution (25%), the solvent was removed in a preheated vacuum centrifuge to a residue of approximately 50 μL. Afterwards, 50 μL of 1% acetic acid was added, and the sample was centrifuged again for 5 min at 16,000 × *g* and transferred to a HPLC vial.

### 2.3 Extraction of Kisspeptin‐14, Kisspeptin‐13, Kisspeptin‐10, and TAK‐448 From Urine Samples

Weak cation‐exchange SPE was also used for the extraction of the smaller kisspeptins from urine samples. Firstly, 1 mL of urine was fortified with 10 μL of d_4_‐Ala‐GHRP‐5 (ISTD 2) solution to a final concentration of 5 ng/mL, which was sufficient given the high analytical sensitivity for this peptide in urine and thoroughly vortexed. HR‐XCW SPE columns were conditioned with 1 mL MeOH and 1 mL water. The urine sample was added, slowly drawn through the column and subsequently washed with 1 mL of water and 1 mL of MeOH. Analytes were eluted with 1 mL of 0.5% TFA in MeOH. After neutralization of the eluate with 4 μL of NH_3_ solution (25%), the solvent was evaporated to a residue of approximately 50 μL, and the samples were reconstituted with 50 μL 1% acetic acid and transferred to HPLC vials.

### 2.4 In Vitro Metabolism Experiments

To examine the stability and metabolization of kisspeptin and its analogues, different in vitro experiments were performed. Therefore, 100 ng of each peptide was incubated in 100 μL of human skin S9 mix, serum, plasma, or urine under gentle shaking (400 rpm) at 37°C for 2 h. Control experiments without substrate (substrate blank) and without enzyme (enzyme blank, EB) were performed in PBS, accordingly. The stability of KP‐54 in urine was not examined due to lack of relevance. Serum, plasma, and urine were prepared by pooling samples collected from six healthy donors (three males, three females). For skin S9 mix, 10 μL of the S9 fraction were added to 90 μL of PBS. To assess analyte stability, additional aliquots were prepared and incubated for 2, 6, and 24 h at 4°C and 37°C, respectively. In addition, freeze–thaw stability was assessed in serum, plasma, and urine over two cycles by repeated freezing (−20°C) and thawing of the samples, with 24‐h storage at −20°C between cycles. For each analyte and each matrix, one aliquot was processed directly and measured as a reference sample (t0). Afterwards, 10 μL of ISTD 2 solution and 400 μL of ACN were added to the samples, and precipitated proteins separated by centrifugation at 16,000 × *g* for 10 min. The supernatant was transferred to a fresh Lo‐Bind Eppendorf tube and evaporated by vacuum‐centrifugation. The residue was reconstituted in 100 μL of 1% acetic acid, followed by another centrifugation step (16,000 × *g*, 5 min) and transferred to an HPLC vial for analysis. All experiments were performed in duplicate.

### 2.5 Liquid‐Chromatography–High‐Resolution Mass Spectrometry

Chromatographic separation of the analytes was accomplished using a Vanquish UHPLC system from Thermo Fisher Scientific (Bremen, Germany). The conditions employed were identical for serum, urine, and in vitro metabolism samples. Eluent A consisted of 0.1% FA and 1% DMSO in water; Eluent B consisted of 0.1% FA and 1% DMSO in ACN. Analytes were first trapped for 2 min on an Accucore Phenyl‐Hexyl column (3 × 10 mm, 2.6 μm, Thermo Fisher Scientific, Bremen, Germany) at 1% B and a flow rate of 0.4 mL/min. Afterwards, the flow was directed to the analytical column, a Poroshell EC‐C18 column (3 × 50 mm, 2.7 μm, Agilent Technologies, Santa Clara, CA, USA). The total runtime was 15 min, and the flow rate was set at 0.4 mL/min. The gradient started isocratic at 5% B for 3 min and then increased to 40% B within 5 min, followed by an increase to 80% B within 2 min as a wash step. Afterwards, the column was re‐equilibrated at starting conditions of 5% B. The injection volume was 15 μL. Mass spectrometric analysis was performed using a Thermo Orbitrap Exploris 480 mass spectrometer employed with a heated electrospray ion source (H‐ESI) operating in positive ionization mode at a voltage of 3000 V. The vaporizer temperature was set at 300°C, the ion transfer tube at 320°C. Sheath gas and auxiliary gas flow rates were set to 30 and 10 arbitrary units, respectively. For confirmatory analysis of kisspeptins in serum and urine samples, targeted MS^2^ experiments were performed. LC‐MS characteristics, including retention times, selected precursor ions, charge states, product ions, and the normalized collision energies (NCE), are listed in Table 1 (KP‐54, ISTD 1) and 2 (KP‐14, KP‐13, KP‐10, TAK‐448, ISTD 2). Precursor ions were isolated with a window of *m*/*z* 3 and the Orbitrap resolution was set at 45,000 FWHM. Additional full scan data were acquired with a scan range of *m*/*z* 200–2000 (resolution: 60,000 FWHM). For detection and identification of peptide metabolites, additional all‐ion fragmentation (AIF) experiments were performed, with an isolation range of *m*/*z* 200–2000 and a scan range of *m*/*z* 200–1000 (resolution: 60,000 FWHM, NCE: 25%). Individual MS^2^ experiments with an isolation window of *m*/*z* 2 and individually optimized collision energies were performed for structure elucidation and amino acid sequencing (resolution: 30,000 FWHM).

**TABLE 1:** LC‐HRMS/MS characteristics for the analysis of KP‐54 in human serum and the analysis of KP‐14, KP‐13, KP‐10, and TAK‐448 in human urine samples.

Analyte	RT (min)	Precursor ion (*m*/*z*)	*z*	NCE (%)	Product ions (*m*/*z*)
KP‐54	6.1	976.6713	6+	23	1083.3600 (y_49_^5+^)
					902.7998 (y_49_^6+^)
Rat Amylin (ISTD 1)	6.8	980.4980	4+	30	920.4681 (b_26_^3+^)
					1282.1399 (b_24_^2+^)
KP‐14	7.2	871.4259	2+	35	839.4528 (y_7_^+^)
					1139.5757 (y_9_^+^)
KP‐13	7.0	813.9124	2+	35	839.4528 (y_7_^+^)
					1139.5757 (y_9_^+^)
KP‐10	6.8	651.8225	2+	30	839.4528 (y_7_^+^)
					725.4092 (y_6_^+^)
TAK‐448	7.0	1225.6113	1+	22	513.2935 (x_3_^+^)
					487.3144 (y_3_^+^)
d_4_‐Ala‐GHRP‐5 (ISTD 2)	8.0	775.3864	1+	30	350.1505 (b_2_^+^)
					425.2127 (b_3_^+^)

Abbreviations: NCE, normalized collision energy; RT, retention time.

### 2.6 Data Evaluation and Visualization

LC‐MS data were processed using TraceFinder (Version 5.2, Thermo Fisher Scientific). For the identification of potential peptide metabolites, the raw data obtained from the in vitro metabolism experiments were processed using MZmine (Version 4.7.8), including mass detection, chromatogram building, deconvolution, alignment, gap filling, and blank subtraction [26]. To reduce redundancy, the resulting feature lists were further filtered to identify and remove features corresponding to the same peptide, including isotopologues, in‐source fragments, and different ion species (i.e., adducts or alternative charge states). Based on the most abundant intact masses identified here, further MS^2^ experiments were performed to enable sequencing and further structural elucidation of putative metabolites.

### 2.7 Method Validation

Both methods, the analysis of KP‐54 in serum and the detection of the smaller kisspeptins in urine samples, were validated according to the recommendations for qualitative confirmation procedures provided by the WADA International Standard for Laboratories (ISL) [27]. These include the evaluation of selectivity, limit of identification (LOI), robustness, carryover, and sample extract stability. For selectivity, 10 different serum samples (or urine samples, as applicable) were processed as described above and analyzed for possible interferences. The LOI was determined by spiking six different samples, each at a minimum of four different concentration levels, and calculating the identification rate for each level. For identification, the LC‐MS criteria specified by the respective WADA technical document were employed, and the LOI was set at a 95% identification rate. The influence of the pH during evaporation on the robustness of the method was investigated by omitting the neutralization of six samples spiked at the minimum required performance level (MPRL) as defined by the corresponding WADA technical document (2 ng/mL) after elution from the SPE columns. In addition, the replacement of SPE columns with Strata X‐CW (1 mL/30 mg, 33 μm) from Phenomenex (Aschaffenburg, Germany) was evaluated, along with chromatography using DMSO‐free eluents (A + B), to further assess the robustness of the method. Analytical carryover was assessed at a concentration of 20 ng/mL by subsequent analysis of a blank sample. Sample extract stability was evaluated after storage for 72 h in the autosampler (+10°C). Additionally, 10 samples were fortified with increasing amounts of the analytes, and linear regression analysis was performed. The recovery rate was determined for all analytes by comparing samples spiked with 2 ng/mL of analyte before and after the respective extraction procedure. For all spiked samples, aliquoted working solutions (0.1 mg/mL) were freshly thawed and diluted with water immediately prior to use. Calibrator samples were prepared by fortifying blank serum (KP‐54) or blank urine with the respective stock solution, followed by serial dilution with the corresponding blank matrix to obtain the desired concentrations in a final volume of 0.2 mL (serum) and 1 mL (urine), respectively.

### 2.8 Reference Population

As a reference population for endogenous KP‐54 concentrations and to evaluate the potential presence of KP‐54 metabolites, 20 serum samples were processed as described above and subjected to LC‐MS analysis. These samples included specimens that were routinely analyzed in doping controls with negative test results, encompassing *n* = 10 female and *n* = 10 male athletes. Similarly, a reference population consisting of *n* = 50 male and *n* = 50 female urine samples from routine doping controls was examined for the presence of KP‐14, KP‐13, and KP‐10, as well as their degradation products, following the protocol described above (100 samples in total). All samples included here had documented research consent and were analyzed in anonymized form in accordance with applicable anti‐doping and ethical standards.

## 3 Results and Discussion

### 3.1 Method Validation

Renal clearance of peptide hormones is hardly predictable, and whereas urinary detection of peptides of comparable molecular size, for example, insulin analogues, has been reported in the literature, the renal elimination of KP‐54 has not been characterized to date [28]. However, due to glomerular filtration, peptides of lower molecular mass are generally more likely to be excreted in urine [29]. Considering the higher molecular mass of KP‐54 and potentially limited glomerular filtration and/or extensive renal degradation, serum analysis was performed for KP‐54, whereas urine analysis was applied to peptides with molecular masses < 2 kDa. The extraction method employed in this study to determine KP‐54 in human serum samples, as well as KP‐14, KP‐13, KP‐10, and TAK‐448 in urine samples, based on mixed‐mode weak cation‐exchange SPE, proved to be highly suitable for a selective and sensitive analysis of all kisspeptins by LC‐MS. A summary of the method validation results for the analysis in serum and urine is shown in Tables 2 and 3, respectively.

**TABLE 2:** Method validation results for the determination of KP‐54 in human serum samples.

Parameter	*n*	c (ng/mL)	Result
Selectivity	10	—	✓
LOI	6 per level	0.1; 0.2; 0.5; 1	0.8 ng/mL
Robustness (pH 2)	6	2	
Bias RRT			0%
Bias response			−10%
Robustness (Strata X‐CW)	6	2	
Bias RRT			0%
Bias response			56%
Robustness (w/o DMSO)	6	2	
Bias RRT			−1%
Bias response			−65%
Carryover	1	20	0%
72‐h sample extract stability	6 + 6	2	98%–126%
Recovery	6 + 6	2	41%–60%
Linearity	10	1–20	*R* ^2^ = 0.9937

Abbreviation: RRT, relative retention time.

**TABLE 3:** Method validation results for the determination of KP‐14, KP‐13, KP‐10, and TAK‐448 in human urine samples.

Parameter	*n*	c (ng/mL)	KP‐14	KP‐13	KP‐10	TAK‐448
Selectivity	10	—	✓	✓	✓	✓
LOI	6 per level	0.01, 0.02, 0.05, 0.1, 0.2	0.2 ng/mL	0.1 ng/mL	0.05 ng/mL	0.01 ng/mL
Robustness (pH 2)		2				
Bias RRT			0%	0%	0%	0%
Bias response			−28%	−7%	−40%	+4%
Robustness (Strata X‐CW)	6	2				
Bias RRT			0%	0%	0%	0%
Bias response			3%	2%	33%	−7%
Robustness (w/o DMSO)	6	2				
Bias RRT			1%	1%	1%	0%
Bias response			−72%	−70%	−72%	−73%
Carryover	1	20	0%	1%	0%	0%
72‐h sample extract stability	6 + 6	2	63%─114%	68%─124%	65%─101%	70%─93%
Recovery	6 + 6	2	58%─97%	80%─100%	71%─100%	54%─100%
Linearity	9	1─15	*R* ^2^ = 0.9942	*R* ^2^ = 0.9958	*R* ^2^ = 0.9975	*R* ^2^ = 0.9912

Abbreviation: RRT, relative retention time.

**FIGURE 2:**
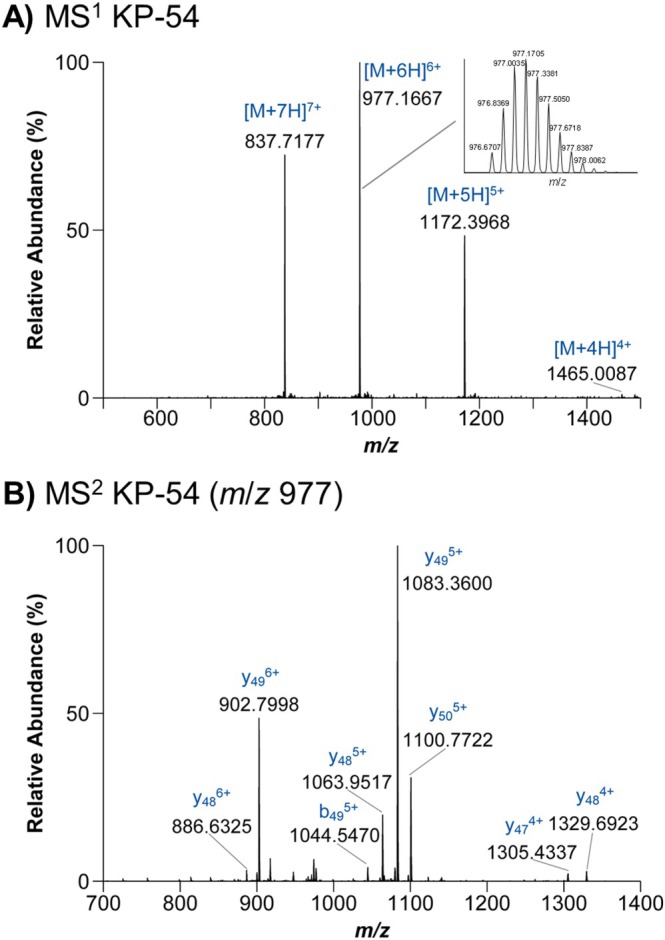
Full scan mass spectrum of KP‐54 (A) and corresponding product ion mass spectrum (B) of the precursor ion selected at *m*/*z* 977, NCE: 23%.

For MS^2^ analysis of KP‐54, the sixfold protonated precursor ion was selected, and the peptide could be confidently identified at a concentration of 0.8 ng/mL employing two selective ion transitions. Representative MS^1^ and MS^2^ spectra are presented in Figure 2. Representative chromatograms of a blank serum sample and a sample spiked at the LOI are provided in Figure S1. Based on existing approaches for peptide screening in doping control blood samples, a purification strategy employing mixed‐mode anion‐exchange cartridges was initially evaluated; however, this resulted in low recovery rates [30]. Owing to the basic properties of the peptide (theoretical pI: 10.44), high selectivity and recovery were achieved utilizing cation‐exchange SPE. Interestingly, the binding of the peptide was very strong with respect to the weak cation exchange capacity of the SPE sorbent. Thus, even at high concentrations, elution could not be achieved using standard protocols with solutions (1%–5%) of ammonia or formic acid in methanol. Only the use of TFA as a modifier in MeOH resulted in good recovery, which can likely be attributed to the ion pair formation properties of the reagent [31]. However, acid‐catalyzed deamidation of KP‐54 was observed, already when using 0.5% TFA only, requiring the samples to be neutralized timely with ammonia following elution. The necessity of this measure was examined in the course of method robustness evaluation, and it could be shown that, although deamidation causes peak broadening, omitting neutralization only leads to a 10% decrease in response. The use of Strata X‐CW SPE columns proved to be straightforward and can even result in improved extraction efficiency, without affecting the analysis as such. Analysis with DMSO‐free eluents resulted in a 65% reduction of mean absolute response, indicating the beneficial effect of DMSO on ionization and analytical sensitivity; however, analysis without DMSO is feasible in principle. At a concentration as high as 20 ng/mL, no analytical carryover was observed, and the sample extracts remained stable over 72 h in the autosampler at +10°C.

**FIGURE 3:**
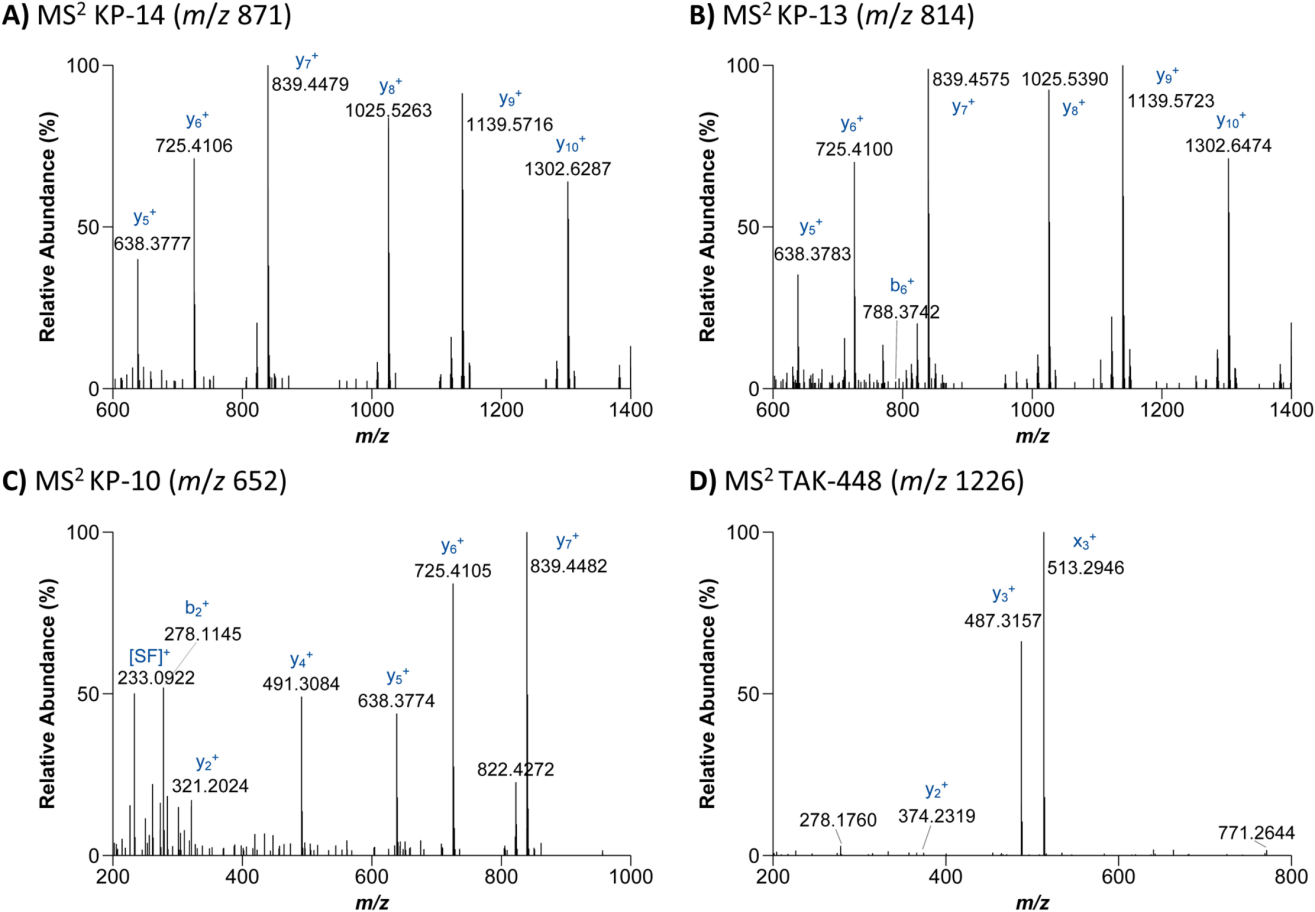
Product ion mass spectra generated by higher energy collision‐induced dissociation of (A) KP‐14, NCE: 35%; (B) KP‐13, NCE: 35%; (C) KP‐10, NCE: 30%; and (D) TAK‐448, NCE: 22%.

For the lower molecular mass peptides in urine, MS^2^ analysis enabled LOIs of ≤ 200 pg/mL; for the KISS1R agonist TAK‐448, a particularly good sensitivity was achieved (LOI: 10 pg/mL). This can partially be attributed to the superior stability of the synthetic peptide, as well as to the specific higher‐energy collision‐induced dissociation (HCD) behavior of the peptide. Representative product ion mass spectra of all peptides analyzed in urine are shown in Figure 3. Chromatograms of a blank urine sample and a urine sample spiked at the LOI of each target analyte can be found in Figure S2. In contrast to the natural peptides, which generate MS^2^ spectra comprising multiple y‐ and b‐ions, the spectrum of TAK‐448 was dominated by two intense product ions originating from dissociation at the (non‐natural) Phe5‐aza‐Gly6 bond. Evaluating selectivity, despite the endogenous occurrence of the peptides, no interfering signals were detected in the 10 urine samples analyzed. The influence of TFA on the analysis of the peptides varied. A significant reduction in response was observed for KP‐14 and KP‐10, indicating that the neutralization of the SPE eluates should be ensured. The use of Strata X‐CW SPE columns did not negatively impact relative retention times (RRT) or absolute response. However, similar to KP‐54, omission of DMSO resulted in a decrease in sensitivity of approximately 70% for all analytes. Unlike in serum, plasma, or urine, the stability of the extracted peptides at +10°C in the autosampler was sufficient over 72 h to ensure reliable analysis.

### 3.2 Stability in Serum, Plasma, and Urine

**FIGURE 4:**
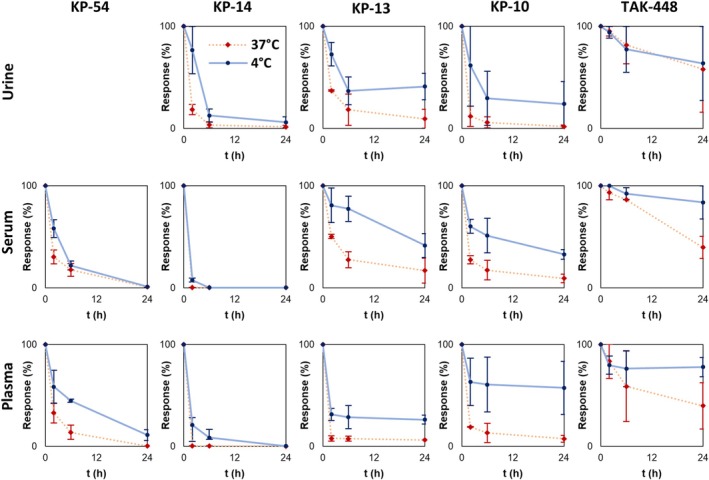
Stability of kisspeptin and its analogues in urine, serum, and plasma depicted as percentage response relative to the starting point (*t* = 0 h). Presentation of average values; error bars indicate maximum and minimum values (*n* = 2).

In view of the partial instability of endogenous kisspeptins observed in clinical studies, incubation experiments were conducted for all analytes at 37°C (simulating body temperature) and at 4°C (simulating storage temperature) in urine, serum, and plasma. Figure 4 shows the percentage response (target analyte/ISTD) in relation to the starting point (*t* = 0 h) after 6 and 24 h in the different matrices and under the different storage conditions. The average values from both experiments are shown, as well as the maximum and minimum values as error bars. The results show that KP‐14 degrades very rapidly in both blood and urine, only slightly affected by reduced temperature. The other peptides show marginally better stability, especially when stored at 4°C. In comparison, the stability of the synthetic peptide TAK‐448 is significantly better, which can be attributed to the modified amino acids (hydroxy‐Pro, aza‐Gly, and methyl‐Arg), which are less accessible to proteases [16]. It should be mentioned that, even though freshly obtained blood and urine samples were used, the incubation experiments conducted here do not necessarily reflect actual in vivo conditions (influence of adsorption processes, lack of clearance, lack of transport, and tissue uptake). Nevertheless, the results confirm the rapid proteolysis of endogenous kisspeptins described in the literature. This applies not only to blood but also to urine samples, which could compromise the ability to detect the misuse of kisspeptin and its analogues for doping purposes.

In addition to short‐term stability at 37°C and 4°C, freeze–thaw stability was investigated, and the results are summarized in Table 4. Although repeated freezing and thawing did not significantly affect the response of TAK‐448, a clear decline was observed for all endogenous kisspeptins. A single freeze–thaw cycle still yielded acceptable results; however, a second cycle resulted in a pronounced loss of signal. This effect was particularly evident for KP‐13, which retained only 9%–19% of its relative response, depending on the matrix, compared to the initial value. These findings suggest that repeated freeze–thaw cycles should be avoided for these analytes to prevent substantial analyte degradation or loss.

**TABLE 4:** Freeze–thaw stability of KP‐54, KP‐14, KP‐13, KP‐10, and TAK‐448 in serum, plasma, and urine, expressed as mean relative response (% of day 0) ± standard deviation (SD).

Matrix	Analyte	Cycle 1 (%)	Cycle 2 (%)
Serum	KP‐54	90 ± 10	46 ± 0
	KP‐14	66 ± 2	15 ± 0
	KP‐13	40 ± 8	19 ± 14
	KP‐10	54 ± 3	33 ± 4
	TAK‐448	88 ± 2	96 ± 3
Plasma	KP‐54	88 ± 11	57 ± 4
	KP‐14	108 ± 24	45 ± 6
	KP‐13	59 ± 14	9 ± 1
	KP‐10	83 ± 14	32 ± 5
	TAK‐448	116 ± 3	95 ± 4
Urine	KP‐14	84 ± 24	34 ± 1
	KP‐13	56 ± 14	12 ± 0
	KP‐10	92 ± 2	56 ± 0
	TAK‐448	111 ± 1	79 ± 3

In general, many peptide hormones and small peptides that are misused for doping purposes have short in vivo half‐lives and tend to undergo oxidation and enzymatic proteolysis [32]. Strict control during the collection, transport, and storage of doping control samples is therefore essential to minimize degradation, microbial growth, and hemolysis. Current WADA guidelines do, however, not mandate active cooling of urine samples, even when transport may take several days [33]. As the absence of cooling can markedly accelerate analyte degradation and microbial growth, our findings highlight the importance of temperature‐controlled transport and storage of doping control samples to ensure reliable analysis of peptides such as kisspeptins. Based on the experiments conducted here, freezing at −20°C, or at least refrigeration at +4°C, and the avoidance of repeated freezing and thawing appears appropriate to prevent peptide degradation in doping control blood or urine samples. Dried blood spots (DBS) may offer improved stability while simplifying transport and storage; they should therefore also be considered for kisspeptins [34]. Another way to enhance detectability could be to (complementary) target degradation products, that is, metabolites of the peptides in doping control analysis. Thus, this study included a comprehensive investigation of the metabolism of all endogenous kisspeptins and TAK‐448 in various biological fluids.

### 3.3 In Vitro Metabolism Experiments

Consistent with the rapid degradation of KP‐54 and the smaller kisspeptins in blood and urine, a large number of metabolites could be identified for all analytes. The detection of potential peptide degradation products was achieved in an untargeted approach using the background subtraction feature of MZmine. Based on the intact masses obtained, MS^2^ experiments were then performed to determine the amino acid sequences and possible modifications. The 10 most abundant metabolites of all endogenous kisspeptins and TAK‐448 are listed in Tables 5, 6, 7, 8, 9. Signal intensities were classified semiquantitatively into three categories (“+,” “++,” and “+++”), corresponding to increasing signal levels (< 1 × 10^6^, ≥ 1 × 10^6^, and ≥ 1 × 10^7^, respectively). Classification was based on the maximum signal observed across two independent experiments (*n* = 2). Product ion mass spectra of all metabolites presented here are included in Figures S3–S52. For KP‐54, a predominant cleavage was observed between Arg25 and Gln26, with both the intact C‐terminal (KP‐54 M7) and intact N‐terminal fragments (KP‐54 M1) being identified. A metabolite of the same sequence as M7 was found but comprising a terminal pyroglutamate (KP‐54 M8), resulting from N‐terminal cyclization of glutamine, which can occur both enzymatically or by a spontaneous loss of ammonia (mass difference −17 Da) [35, 36]. Analogous to the processes described for human circulation, cleavage to the bioactive fragment KP‐14 was observed, too (KP‐54 M9).

**TABLE 5:** Major degradation products identified for KP‐54 in serum, plasma, and skin S9 fraction. Maximum signal intensity across *n* = 2 experiments categorized as follows: + < 1 × 10^6^; ++ ≥ 1 × 10^6^; +++ ≥ 1 × 10^7^.

#	RT	Precursor ion (*m*/*z*)	*z*	Proposed sequence	Position	C‐terminus	Modification	Serum	Plasma	Skin S9	EB (PBS)
M1	4.29	839.7529	3+	GTSLSPPPESSGSRQQPGLSAPHSR	1–25	Free acid		**+**		**++**	**+**
M2	4.53	787.7192	3+	GTSLSPPPESSGSRQQPGLSAPHS	1–24	Free acid		**++**	**+**	**+**	
M3	5.43	898.4723	4+	GTSLSPPPESSGSRQQPGLSAPHSRQIPAPQGAVLV	1–36	Free acid		**+**	**++**		
M4	6.36	900.73	4+	SRQIPAPQGAVLVQREKDLPNYNWNSFGLRF	24–54	Amide		**++**	**+**	**+**	
M5	6.57	570.2909	2+	NWNSFGLRF	46–54	Amide		**++**	**++**		
M6	6.62	798.3837	2+	DLPNYNWNSFGLR	41–53	Free acid			**+**	**++**	
M7	6.64	1119.5932	3+	QIPAPQGAVLVQREKDLPNYNWNSFGLRF	26–54	Amide		**+++**	**+**		
M8	7.04	1113.9177	3+	pE*IPAPQGAVLVQREKDLPNYNWNSFGLRF	26–54	Amide	Q1 ↔ pE	**+++**	**++**		
M9	7.2	871.4259	2+	DLPNYNWNSFGLRF	41–54	Amide		**++**	**++**		
M10	7.24	1519.2802	2+	pE*IPAPQGAVLVQREKDLPNYNWNSFGL	26–52	Free acid	Q1 ↔ pE	**+++**	**++**	**+**	

Abbreviations: EB, Enzme blank; pE, pyroglutamate.

**TABLE 6:** Major degradation products identified for KP‐14 in serum, plasma, urine and skin S9 fraction. Maximum signal intensity across *n* = 2 experiments categorized as follows: + < 1 × 10^6^; ++ ≥ 1 × 10^6^; +++ ≥ 1 × 10^7^.

#	RT	Precursor ion (*m*/*z*)	*z*	Proposed sequence	Position	C‐terminus	Modification	Serum	Plasma	Skin S9	Urine	EB (PBS)
M1	5.71	724.3049	1+	NWNSFG	6–11	Free acid		**++**	**++**	**++**		
M2	5.89	839.4522	1+	NSFGLRF	8–14	Amide		**++**	**++**	**+++**	**+**	**+**
M3	5.99	725.4093	1+	SFGLRF	9–14	Amide		**++**	**++**	**+++**	**+**	**+**
M4	6.09	840.4363	1+	DSFGLRF	8–14	Amide	N1 ↔ D	**++**	**++**		**+**	
M5	6.35	636.2938	2+	DYNWNSFGLR	4–13	Free acid	N1 ↔ D				**+++**	
M6	6.49	740.8702	2+	LPNYNWNSFGLR	2–13	Free acid		**++**		**++**	**++**	
M7	6.5	513.2694	2+	WNSFGLRF	7–14	Amide		**+**	**+**	**+++**		
M8	6.67	798.3837	2+	DLPNYNWNSFGLR	1–13	Free acid		**++**	**+**	**+++**	**+++**	
M9	6.68	570.2909	2+	NWNSFGLRF	6–14	Amide		**++**	**++**	**+++**		
M10	7.03	813.9124	2+	LPNYNWNSFGLRF	2–14	Amide		**++**	**+**	**++**	**+**	

Abbreviation: EB, Enzme blank.

**TABLE 7:** Major degradation products identified for KP‐13 in serum, plasma, urine, and skin S9 fraction. Maximum signal intensity across *n* = 2 experiments categorized as follows: + < 1 × 10^6^; ++ ≥ 1 × 10^6^; +++ ≥ 1 × 10^7^.

#	RT	Precursor ion (*m*/*z*)	*z*	Proposed sequence	Position	C‐terminus	Modification	Serum	Plasma	Skin S9	Urine	EB (PBS)
M1	4.88	711.2733	1+	DYNWN	3–7	Free acid	N1 ↔ D				**+**	
M2	5.71	724.3049	1+	NWNSFG	5–10	Free acid		**+**	**+**	**++**		
M3	5.81	839.4522	1+	NSFGLRF	7–13	Amide		**++**	**+**	**++**		**+**
M4	5.9	725.4093	1+	SFGLRF	8–13	Amide				**++**		
M5	6.31	636.2938	2+	DYNWNSFGLR	3–12	Free acid	N1 ↔ D	**+**		**+**	**+**	
M6	6.38	945.3737	1+	DYNWNSF	3–9	Free acid	N1 ↔ D			**+**	**+**	
M7	6.46	1025.5316	1+	WNSFGLRF	6–13	Amide		**+**		**+**	**+**	
M8	6.53	740.8702	2+	LPNYNWNSFGLR	1–12	Free acid				**+**	**+**	
M9	6.59	570.2909	2+	NWNSFGLRF	5–13	Amide		**++**	**+**	**+**		
M10	6.82	651.8225	2+	YNWNSFGLRF	4–13	Amide		**++**		**+++**		**+**

Abbreviation: EB, Enzme blank.

**TABLE 8:** Major degradation products identified for KP‐10 in serum, plasma, urine, and S9 fraction. Maximum signal intensity across *n* = 2 experiments categorized as follows: + < 1 × 10^6^; ++ ≥ 1 × 10^6^; +++ ≥ 1 × 10^7^.

#	RT	Precursor ion (*m*/*z*)	*z*	Proposed sequence	Position	C‐terminus	Modification	Serum	Plasma	Skin S9	Urine	EB (PBS)
M1	5.74	752.4202	1+	NFGLRF	5–10	Amide	S1 ↔ N	**++**		**+**		
M2	5.82	839.4522	1+	NSFGLRF	4–10	Amide		**+++**	**++**	**+++**	**+**	**+**
M3	5.9	725.4093	1+	SFGLRF	5–10	Amide		**+++**	**++**			
M4	6.19	578.7803	2+	YNWNSFGLR	1–9	Free acid		**++**	**+**	**+++**	**+++**	
M5	6.19	840.4363	1+	DSFGLRF	4–10	Amide	N1 ↔ D	**+++**	**++**		**++**	
M6	6.28	830.3468	1+	YNWNSF	1–6	Free acid		**+**		**+++**	**+++**	**++**
M7	6.6	570.2909	2+	NWNSFGLRF	2–10	Amide		**+++**	**+++**	**+++**	**+**	**+**
M8	6.82	652.3146	2+	YNWNSFGLRF	1–10	Free acid		**++**	**+**	**+++**	**+**	**+**
M9	7.08	652.8066	2+	YDWDSFGLRF	1–10	Free acid	N2 ↔ D	**++**	**+**	**++**		
M10	7.11	1000.4523	1+	YNWNSFGL	1–8	Free acid		**++**		**+++**	**+++**	**+**

Abbreviation: EB, Enzme blank.

**TABLE 9:** Major degradation products identified for TAK‐448 in serum, plasma, urine, and S9 fraction. Maximum signal intensity across *n* = 2 experiments categorized as follows: + < 1 × 10^6^; ++ ≥ 1 × 10^6^; +++ ≥ 1 × 10^7^.

#	RT	Precursor ion (*m*/*z*)	*z*	Proposed sequence	Position	C‐terminus	Modification	Serum	Plasma	Skin S9	Urine	EB (PBS)
M1	4.89	430.2409	1+	[C_3_H_2_O_2_‐aza]GL[methyl]R	6–8	Free acid	azaG1 + C_3_H_2_O_2_	✓		✓		
M2	5.59	545.3307	1+	[aza]GL[methyl]RW	6–9	Amide		✓	✓		✓	✓
M3	5.83	692.3991	1+	F[aza]GL[methyl]RW	5–9	Amide		✓	✓	✓	✓	
M4	5.85	793.4468	1+	TF[aza]GL[methyl]RW	4–9	Amide		✓	✓	✓	✓	✓
M5	5.98	699.2984	1+	Ac‐Y[OH]PNTF	1–5	Free acid		✓	✓	✓	✓	✓
M6	6.12	1040.5160	1+	Ac‐Y[OH]PNTF[aza]GL [methyl]R	1–8	Free acid		✓	✓	✓	✓	✓
M7	6.46	615.3362	1+	[C_3_H_2_O_2_‐aza]GL [methyl]RW	6–9	Amide	azaG1 + C_3_H_2_O_2_	✓	✓	✓	✓	✓
M8	6.57	557.3307	1+	[C_1_‐aza]GL[methyl]RW	6–9	Amide	azaG1 + C_1_	✓	✓		✓	✓
M9	6.90	571.3463	1+	[C_2_H_2_‐aza]GL[methyl]RW	6–9	Amide	azaG1 + C_2_H_2_	✓			✓	
M10	7.36	1226.5953	1+	Ac‐Y[OH]PNTF[aza]GL [methyl]RW	1–9	Free acid		✓	✓	✓	✓	✓

Abbreviation: EB, Enzme blank.

The metabolites of KP‐14 and KP‐13 showed many overlaps, which can be expected given the difference of only one amino acid between the two peptides. In each case, several metabolites with intact C‐termini could be identified. For KP‐13, a predominant cleavage was observed between Pro2 and Asn3, followed by deamidation to Asp (KP‐13 M1, M5, and M6). Here, too, further proteolytic cleavage to KP‐13 (KP‐14 M10) and to KP‐10 (KP‐13 M10) was observed.

For KP‐10, it was observed that the characteristic C‐terminus containing the Arg‐Phe‐NH_2_ motif remained intact in the most abundant degradation products. Previously, the metabolism of KP‐10 in serum has been investigated by Colpaert et al., who already described the metabolites KP‐10 M2 and M7 [22]. The latter, an N‐terminal tyrosine‐depleted peptide (*m*/*z* 570.3 Da), was also identified in rat plasma in a 2013 study investigating the degradation of KP‐10 post‐administration [13]. Although this was not a human study, this metabolite—representing a common degradation product of all endogenous kisspeptins—has thus been detected in vivo and can be considered biologically relevant. An oxidation product described by Colpaert et al. in a KP‐10 standard, arising from the oxidation of tryptophan to kynurenine, was not detected in the present experiments [22]. Instead, N‐terminally deamidated KP‐10 (free acid) as well as a form with twofold deamidation (KP‐10 M9) were identified.

**FIGURE 5:**
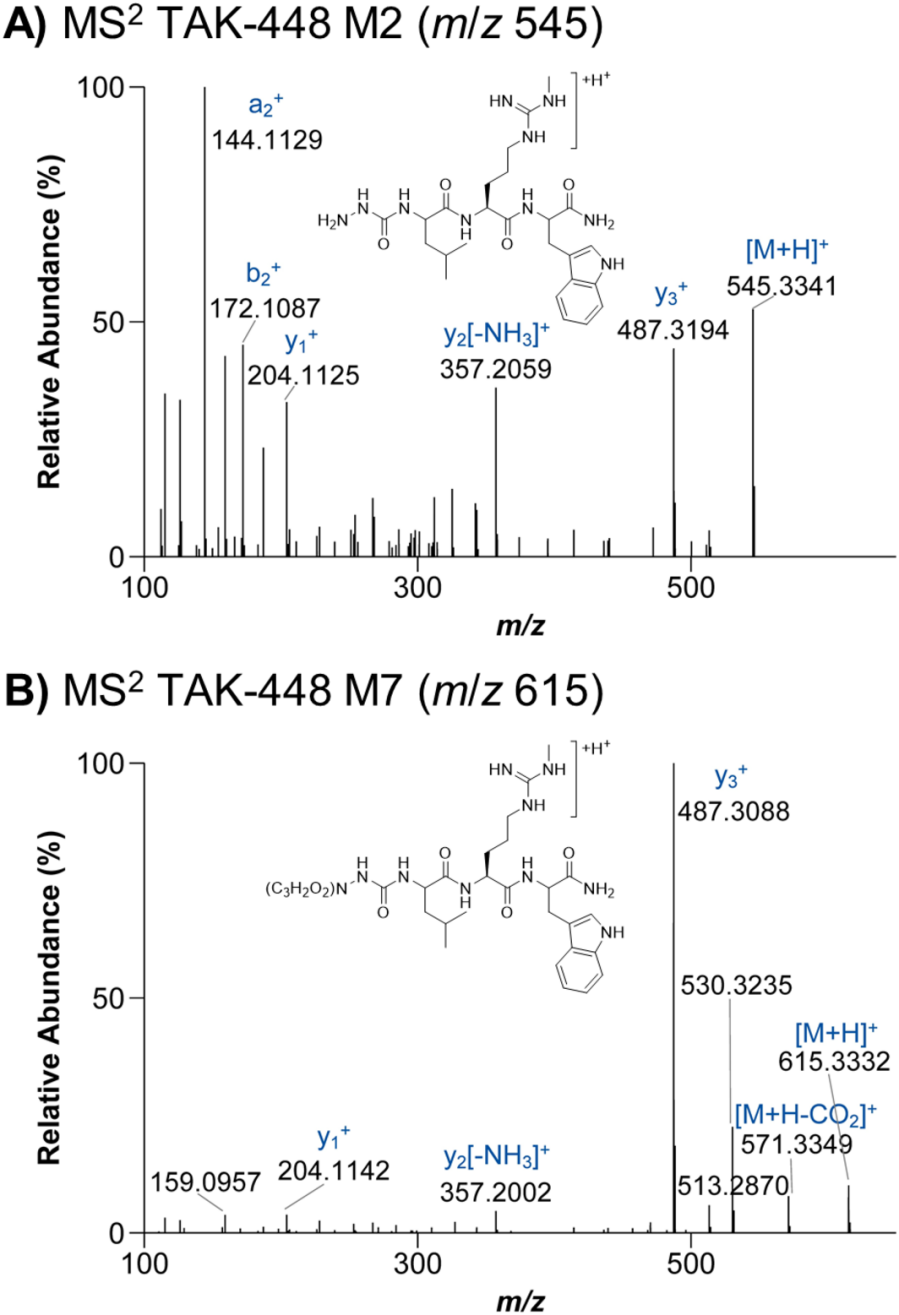
Product ion mass spectra of (A) TAK‐448 M2, precursor ion *m*/*z* 545 (NCE: 35%) and (B) TAK‐448 M7, precursor ion *m*/*z* 615 (NCE: 25%).

As described above, TAK‐448 proved to be significantly more stable than the endogenous kisspeptins, which presumably improves the chance of detecting the intact compound in urine samples after application of the peptide drug. Nevertheless, several metabolites could be identified, including primary proteolytic degradation products such as the metabolites TAK‐448 M3–M6 and N‐terminally deamidated TAK‐448 (free acid, TAK‐448 M10). However, the most intense metabolites in all matrices were those formed by cleavage at the Phe5‐aza‐Gly6 bond. Generally, incorporation of an aza‐Gly residue alters the conformation of the peptide bond and is expected to enhance enzymatic and thermal stability [37, 38]. Therefore, it remains unclear why cleavage appears to occur predominantly at this position. Figure 5A exemplarily shows the product ion mass spectrum of TAK‐448 M2, with the characteristic y_3_^+^ ion (*m*/*z* 487.3). This ion was also highly abundant in the MS^2^ spectra of the metabolites TAK‐448 M1 and M7–M9, which appear to show additional modifications at the aza‐Gly residue. On the basis of exact mass data and diagnostic product ion mass spectra, the metabolites M8 and M9 are consistent with the formation of a N‐terminal‐methylidene functionality (M8) and a N‐terminal vinyl substitution (M9), respectively. Correspondingly, the metabolites TAK‐448 M1 and M7 are consistent with formal N‐terminal vinylation and N‐terminal carboxylation. The product ion spectrum of TAK‐448 M7 is exemplarily shown in Figure 5B.

The results presented here are largely consistent with a recently published study by Tian et al., in which the metabolic profile of TAK‐448 was investigated in human liver microsomes [39]. Tian et al. proposed the unusual biotransformation pathway involving N‐terminal vinylation and N‐terminal carboxylation and suggested a major contribution of CYP450 enzymes, supported by experiments employing CYP enzyme inhibitors. Although the HCD fragmentation patterns are consistent with these assignments, relevant concentrations of the metabolites TAK448 M7 and M8 were also detectable in the enzyme‐free control samples (PBS) within our study. This observation suggests that their formation may, at least in part, occur via nonenzymatic pathways. Alternatively, based on the exact mass and product ion mass spectra of M1 and M7, hydrazone formation with pyruvic acid and potential stabilization via azo‐hydrazone tautomerism is also plausible. This potential structure of M7 is shown as an example in the supplemental information Figure S53. However, studies on the fate of aza‐Gly‐containing peptides are scarce, and it should be emphasized that all structural considerations are based on the chromatographic behavior and acquired MS^2^ spectra only. Definitive structural confirmation can only be achieved through chemical synthesis and subsequent comprehensive characterization. Importantly, the metabolites identified by Tian et al. in vitro were verified by an excretion study with rats, where the peptide was administered either orally or by subcutaneous (s.c.) injection. Within these investigations, the metabolites TAK‐448 M1, M2, and M7 in particular showed a significantly longer detection window (up to 48 h) in urine compared to the parent compound (up to 4 h) after s.c. administration of TAK‐448. Tian et al. reported another metabolite with prolonged detectability (*m*/*z* 552.2) that was not observed within our experiments but had previously been detected by Moriya et al. in the urine and feces of rats and dogs after administration of radioactively labeled TAK‐448 [40]. Interestingly, in this study, the parent compound was not detectable after 24‐h post‐administration, whereas the hydrolyzed metabolite remained detectable. On the one hand, differences observed between our results, that is, the identified metabolites, and the in vivo studies mentioned here can be attributed to methodological differences. Tian et al. employed an experimental setup using human liver microsomes and focused their analysis on metabolites generated within that specific system [39]. However, general limitations of our applied in vitro system may further restrict its capability to fully capture the complexity of human in vivo biotransformation. The reproducibility of the experiments conducted here may be constrained by the inherent complexity of biological matrices. Serum, plasma, urine, and S9 fractions are subject to significant inter‐batch and inter‐donor variability, affecting enzyme composition, protease activity, and, consequently, metabolite formation. In this context, samples obtained after administration of the respective substances are of exceptional value, in order to assess the relevance of the metabolites identified in vitro, as well as the extent and rate of their formation. Overall, the studies by Tian et al. and Moriya et al. underscore the general relevance of investigating peptide metabolism [39, 40]. Such approaches remain essential for improving the detectability of peptide‐based drugs in doping control samples.

### 3.4 Reference Population

A total of *n* = 20 serum samples was analyzed for endogenous signals of KP‐54 employing the extraction procedure described above. Furthermore, samples were screened for metabolites of KP‐54 identified in vitro, as listed in Table 5. In none of the analyzed samples, endogenous KP‐54 or its metabolites were detectable. Reported kisspeptin concentrations in human serum or plasma vary widely across studies, spanning from < 2 pmol/L (i.e., ~12 pg/mL) to low level ng/mL in healthy adults, with a strong influence of age, sex, and cycle phase [24, 41, 42, 43]. However, interpretation of these data is substantially limited by methodological heterogeneity. The majority of published studies rely on radioimmunoassays or enzyme‐linked immunosorbent assays (ELISA), measuring “total kisspeptin” immunoreactivity rather than a defined molecular species. Thus, the reported concentrations reflect an aggregated signal of circulating isoforms with unknown relative contributions, and absolute concentrations ranges reported should be interpreted with caution. To the best of our knowledge, quantitative LC‐MS‐based measurements of endogenous KP‐54 are not available, and a direct comparison of isoform‐specific mass spectrometric data is not possible. Nonetheless, based on the available literature, circulating levels of KP‐54 are likely below the LOI specified for our method, and establishing potential threshold levels does not appear necessary. KP‐54 concentrations in circulation appear to increase markedly during pregnancy, which may not be relevant at present, as testosterone‐stimulating peptides are currently prohibited only for male athletes. Notably, however, the WADA Monitoring Program 2026 includes GnRH analogues in females under 18 years of age, suggesting that kisspeptin analysis may also become relevant for female anti‐doping analysis in the future [44]. In this context, the analysis of blood and/or urine samples from pregnant women could represent an interesting avenue for future research to further evaluate endogenous kisspeptin levels. Furthermore, serum levels can also be increased in certain diseases, which should be considered when analyzing serum samples for the peptide [41, 45]. A comparable situation was already observed with the analysis of GnRH in urine samples. Although GnRH is produced endogenously, no detectable concentrations were found in urine, most likely due to its presence in the microcirculation between hypothalamus and pituitary gland only [46].

Similar to the serum samples, a reference population of *n* = 100 urine samples was processed and analyzed for KP‐14, KP‐13, KP‐10, and their degradation products. Here, too, no endogenous signals could be detected. As described above, there is a lack of data that cleanly separates endogenous KP‐14, KP‐13, and KP‐10 concentrations, and urinary data are generally limited. One study examined “total kisspeptin” in urine samples collected from women throughout their menstrual cycle, measuring concentrations averaging 492 pg/mL in the early follicular phase and up to an average of 1105 pg/mL on the 11th day of the cycle [25]. In another study, immunoreactivity was compared between pregnant and nonpregnant women, with mean concentrations of 301 and 80 pmol/L, respectively [23]. In general, urinary concentrations were higher than the serum concentrations determined in parallel. Due to the limitations described above, it again proves difficult to compare absolute values, but based on the large number of urine samples analyzed within our study, endogenous levels of the different kisspeptin isoforms do not appear to be detectable, despite the high sensitivity of the method. Therefore, it does not appear necessary to set particular limits for the analysis of routine doping control urine samples at this stage. The influence of hormonal diseases on urinary kisspeptin levels should, however, be kept in mind. Generally, our investigations are limited by the lack of human samples obtained after administration of kisspeptin or its analogues. Corresponding serum and/or urine samples would be of great interest in order to estimate the concentration ranges and time periods during which the respective analytes or their degradation products are detectable via LC‐MS‐based methods. A systematic evaluation of the most suitable biological matrix for the detection of kisspeptins following administration would be valuable in future studies. Such investigations could elucidate the renal elimination of KP‐54 and whether smaller kisspeptin fragments (KP‐14, KP‐13, or KP‐10) or their potential metabolites may be detectable in serum. In the context of future controlled excretion studies, it should be considered that administration of kisspeptin may affect individual steroid markers in serum or urine as monitored within the steroidal module of the Athlete Biological Passport (ABP) [20, 47]. Consequently, investigating potential changes in the steroid profile may represent a relevant aspect. In view of the considerable instability of kisspeptins in biological matrix, the storage duration and conditions that allow for a reliable analysis would be additional points of interest. Analysis from DBS may represent a promising alternative approach, particularly considering the potentially improved stability of peptides in the dried matrix.

## 4 Conclusion

This study aimed to provide an analytical and metabolic characterization of kisspeptin and its analogues, which are prohibited in professional sports due to their potential for indirect androgen doping. Selective and sensitive LC‐MS‐based methods were developed for the confirmatory analysis of kisspeptin‐54 in serum and for the smaller isoforms, as well as the synthetic KISS1R agonist TAK‐448 in urine. With the exception of TAK‐448, the peptides showed limited stability in biological matrices; however, a substantial number of potential metabolites were identified that may serve as target analytes to improve detectability in doping control samples. Furthermore, no endogenous signals of the peptides were present in serum or urine samples obtained from healthy athletes, which is an important finding, given that relevant endogenous concentrations might complicate the discrimination between natural presence and exogenous administration of the peptides. Collectively, these findings establish an important basis for a reliable and fair analysis of kisspeptins in doping controls. Future investigations involving samples collected after peptide administration appear warranted to further elucidate the pharmacokinetic behavior, detection capability, and detection windows using LC‐MS‐based analytical methods.

## Funding

This work was supported by World Anti‐Doping Agency (241A14MT), Manfred Donike Institute for Doping Analysis, and the Federal Chancellery of the Federal Republic of Germany.

## Conflicts of Interest

The authors declare no conflicts of interest.

## Supporting information


**Figure S1:** Representative extracted ion chromatograms of a blank serum sample (A) and a serum sample spiked with KP‐54 at the limit of identification corresponding to 0.8 ng/mL (B).
**Figure S2:** Representative extracted ion chromatograms of a blank urine sample (A) and a urine sample spiked at the limit of identification corresponding to 0.01 ng/mL (TAK‐448), 0.2 ng/mL (KP‐14), 0.1 ng/mL (KP‐13), and 0.05 ng/mL (KP‐10), respectively (B).
**Figure S3:** Product ion mass spectrum of KP‐54 M1, *m*/*z* 840 (z = 3), NCE: 30%.
**Figure S4:** Product ion mass spectrum of KP‐54 M2, *m*/*z* 788 (z = 3), NCE: 30%.
**Figure S5:** Product ion mass spectrum of KP‐54 M3, *m*/*z* 898 (z = 4), NCE: 30%.
**Figure S6:** Product ion mass spectrum of KP‐54 M4, *m*/*z* 901 (z = 4), NCE: 30%.
**Figure S7:** Product ion mass spectrum of KP‐54 M5, *m*/*z* 570 (z = 2), NCE: 30%.
**Figure S8:** Product ion mass spectrum of KP‐54 M6, *m*/*z* 798 (z = 2), NCE: 30%.
**Figure S9:** Product ion mass spectrum of KP‐54 M7, *m*/*z* 1120 (z = 3), NCE: 30%.
**Figure S10:** Product ion mass spectrum of KP‐54 M8, *m*/*z* 1114 (z = 3), NCE: 30%.
**Figure S11:** Product ion mass spectrum of KP‐54 M9, *m*/*z* 871 (z = 2), NCE: 30%.
**Figure S12:** Product ion mass spectrum of KP‐54 M10, *m*/*z* 1519 (z = 2), NCE: 30%.
**Figure S13:** Product ion mass spectrum of KP‐14 M1, *m*/*z* 724 (z = 1), NCE: 30%.
**Figure S14:** Product ion mass spectrum of KP‐14 M2, *m*/*z* 839 (z = 1), NCE: 30%.
**Figure S15:** Product ion mass spectrum of KP‐14 M3, *m*/*z* 725 (z = 1), NCE: 40%.
**Figure S16:** Product ion mass spectrum of KP‐14 M4, *m*/*z* 840 (z = 1), NCE: 30%.
**Figure S17:** Product ion mass spectrum of KP‐14 M5, *m*/*z* 636 (z = 2), NCE: 30%.
**Figure S18:** Product ion mass spectrum of KP‐14 M6, *m*/*z* 741 (z = 2), NCE: 30%.
**Figure S19:** Product ion mass spectrum of KP‐14 M7, *m*/*z* 513 (z = 2), NCE: 30%.
**Figure S20:** Product ion mass spectrum of KP‐14 M8, *m*/*z* 798 (z = 2), NCE: 30%.
**Figure S21:** Product ion mass spectrum of KP‐14 M1, *m*/*z* 570 (z = 2), NCE: 30%.
**Figure S22:** Product ion mass spectrum of KP‐14 M10, *m*/*z* 814 (z = 2), NCE: 35%.
**Figure S23:** Product ion mass spectrum of KP‐13 M1, *m*/*z* 711 (z = 1), NCE: 30%.
**Figure S24:** Product ion mass spectrum of KP‐13 M2, *m*/*z* 724 (z = 1), NCE: 30%.
**Figure S25:** Product ion mass spectrum of KP‐13 M3, *m*/*z* 839 (z = 1), NCE: 30%.
**Figure S26:** Product ion mass spectrum of KP‐13 M4, *m*/*z* 725 (z = 1), NCE: 40%.
**Figure S27:** Product ion mass spectrum of KP‐13 M5, *m*/*z* 636 (z = 2), NCE: 30%.
**Figure S28:** Product ion mass spectrum of KP‐13 M6, *m*/*z* 945 (z = 1), NCE: 40%.
**Figure S29:** Product ion mass spectrum of KP‐13 M7, *m*/*z* 1026 (z = 1), NCE: 40%.
**Figure S30:** Product ion mass spectrum of KP‐13 M8, *m*/*z* 741 (z = 2), NCE: 30%.
**Figure S31:** Product ion mass spectrum of KP‐13 M9, *m*/*z* 570 (z = 2), NCE: 30%.
**Figure S32:** Product ion mass spectrum of KP‐13 M10, *m*/*z* 652 (z = 2), NCE: 30%.
**Figure S33:** Product ion mass spectrum of KP‐10 M1, *m*/*z* 752 (z = 1), NCE: 30%.
**Figure S34:** Product ion mass spectrum of KP‐10 M2, *m*/*z* 839 (z = 1), NCE: 30%.
**Figure S35:** Product ion mass spectrum of KP‐10 M3, *m*/*z* 725 (z = 1), NCE: 30%.
**Figure S36:** Product ion mass spectrum of KP‐10 M4, *m*/*z* 578 (z = 2), NCE: 30%.
**Figure S37:** Product ion mass spectrum of KP‐10 M5, *m*/*z* 840 (z = 1), NCE: 30%.
**Figure S38:** Product ion mass spectrum of KP‐10 M6, *m*/*z* 830 (z = 1), NCE: 30%.
**Figure S39:** Product ion mass spectrum of KP‐10 M7, *m*/*z* 570 (z = 2), NCE: 30%.
**Figure S40:** Product ion mass spectrum of KP‐10 M8, *m*/*z* 652 (z = 2), NCE: 30%.
**Figure S41:** Product ion mass spectrum of KP‐10 M9, *m*/*z* 653 (z = 2), NCE: 30%.
**Figure S42:** Product ion mass spectrum of KP‐10 M10, *m*/*z* 1000 (z = 1), NCE: 30%.
**Figure S43:** Product ion mass spectrum of TAK‐448 M1, *m*/*z* 430 (z = 1), NCE: 30%.
**Figure S44:** Product ion mass spectrum of TAK‐448 M2, *m*/*z* 545 (z = 1), NCE: 35%.
**Figure S45:** Product ion mass spectrum of TAK‐448 M3, *m*/*z* 692 (z = 1), NCE: 30%.
**Figure S46:** Product ion mass spectrum of TAK‐448 M4, *m*/*z* 793 (z = 1), NCE: 30%.
**Figure S47:** Product ion mass spectrum of TAK‐448 M5, *m*/*z* 699 (z = 1), NCE: 30%.
**Figure S48:** Product ion mass spectrum of TAK‐448 M6, *m*/*z* 1040 (z = 1), NCE: 30%.
**Figure S49:** Product ion mass spectrum of TAK‐448 M7, *m*/*z* 615 (z = 1), NCE: 25%.
**Figure S50:** Product ion mass spectrum of TAK‐448 M8, *m*/*z* 557 (z = 1), NCE: 30%.
**Figure S51:** Product ion mass spectrum of TAK‐448 M9, *m*/*z* 571 (z = 1), NCE: 30%.
**Figure S52:** Product ion mass spectrum of TAK‐448 M10, *m*/*z* 1227 (z = 1), NCE: 35%.
**Figure S53:** Proposed alternative structure of TAK‐448 M7.

## Data Availability

The data that support the findings of this study are available on request from the corresponding author. The data are not publicly available due to privacy or ethical restrictions.
