# Supplementary material for: Analysis and Characterization of Kisspeptin and Its Analogues in Serum and Urine Samples by Liquid Chromatography–High‐Resolution Mass Spectrometry for Doping Control Purposes
Source: Drug Test Anal. 2026 Apr 29;18(7):862–76. doi: 10.1002/dta.70081 (PMC13327143; doi:10.1002/dta.70081)
Supplement: Supplementary file 1 — Figure S1: Representative extracted ion chromatograms of a blank serum sample (A) and a serum sample spiked with KP‐54 at the limit of identification corresponding to 0.8 ng/mL (B). Figure S2: Representative extracted ion chromatograms of a blank urine sample (A) and a urine sample spiked at the limit of identification corresponding to 0.01 ng/mL (TAK‐448), 0.2 ng/mL (KP‐14), 0.1 ng/mL (KP‐13), and 0.05 ng/mL (KP‐10), respectively (B). Figure S3: Product ion mass spectrum of KP‐54 M1, m/z 840 (z = 3), NCE: 30%. Figure S4: Product ion mass spectrum of KP‐54 M2, m/z 788 (z = 3), NCE: 30%. Figure S5: Product ion mass spectrum of KP‐54 M3, m/z 898 (z = 4), NCE: 30%. Figure S6: Product ion mass spectrum of KP‐54 M4, m/z 901 (z = 4), NCE: 30%. Figure S7: Product ion mass spectrum of KP‐54 M5, m/z 570 (z = 2), NCE: 30%. Figure S8: Product ion mass spectrum of KP‐54 M6, m/z 798 (z = 2), NCE: 30%. Figure S9: Product ion mass spectrum of KP‐54 M7, m/z 1120 (z = 3), NCE: 30%. Figure S10: Product ion mass spectrum of KP‐54 M8, m/z 1114 (z = 3), NCE: 30%. Figure S11: Product ion mass spectrum of KP‐54 M9, m/z 871 (z = 2), NCE: 30%. Figure S12: Product ion mass spectrum of KP‐54 M10, m/z 1519 (z = 2), NCE: 30%. Figure S13: Product ion mass spectrum of KP‐14 M1, m/z 724 (z = 1), NCE: 30%. Figure S14: Product ion mass spectrum of KP‐14 M2, m/z 839 (z = 1), NCE: 30%. Figure S15: Product ion mass spectrum of KP‐14 M3, m/z 725 (z = 1), NCE: 40%. Figure S16: Product ion mass spectrum of KP‐14 M4, m/z 840 (z = 1), NCE: 30%. Figure S17: Product ion mass spectrum of KP‐14 M5, m/z 636 (z = 2), NCE: 30%. Figure S18: Product ion mass spectrum of KP‐14 M6, m/z 741 (z = 2), NCE: 30%. Figure S19: Product ion mass spectrum of KP‐14 M7, m/z 513 (z = 2), NCE: 30%. Figure S20: Product ion mass spectrum of KP‐14 M8, m/z 798 (z = 2), NCE: 30%. Figure S21: Product ion mass spectrum of KP‐14 M1, m/z 570 (z = 2), NCE: 30%. Figure S22: Product ion mass spectrum of KP‐14 M10, m/z 814 (z = 2), NCE: 35%. Fi [file DTA-18-862-s001.docx]

**Analysis and characterization of kisspeptin and its analogues in serum and urine samples by liquid chromatography - high resolution mass spectrometry for doping control purposes**

Sophia Krombholz^1^, Linus Korsmeier^1^, Andreas Thomas^1^, Mario Thevis^1,2^

^1^ Institute of Biochemistry, Center for Preventive Doping Research, German Sport University Cologne, Germany

^2^ European Center for Emerging Doping Agents, Cologne, Germany

**Supplemental information**

[**Figure S1**: Representative extracted ion chromatograms of a blank serum sample (A) and a serum sample spiked with KP-54 at the limit of identification corresponding to 0.8 ng/ml (B). 3](#_Toc224658913)

[**Figure S2**: Representative extracted ion chromatograms of a blank urine sample (A) and a urine sample spiked at the limit of identification corresponding to 0.01 ng/ml (TAK-448), 0.2 ng/ml (KP-14), 0.1 ng/ml (KP-13), and 0.05 ng/ml (KP-10), respectively (B). 3](#_Toc224658914)

[**Figure S3**: Product ion mass spectrum of KP-54 M1, *m*/*z* 840 (z = 3), NCE: 30%. 4](#_Toc224658915)

[**Figure S4**: Product ion mass spectrum of KP-54 M2, *m*/*z* 788 (z = 3), NCE: 30%. 4](#_Toc224658916)

[**Figure S5**: Product ion mass spectrum of KP-54 M3, *m*/*z* 898 (z = 4), NCE: 30%. 4](#_Toc224658917)

[**Figure S6**: Product ion mass spectrum of KP-54 M4, *m*/*z* 901 (z = 4), NCE: 30%. 4](#_Toc224658918)

[**Figure S7**: Product ion mass spectrum of KP-54 M5, *m*/*z* 570 (z = 2), NCE: 30%. 4](#_Toc224658919)

[**Figure S8**: Product ion mass spectrum of KP-54 M6, *m*/*z* 798 (z = 2), NCE: 30%. 4](#_Toc224658920)

[**Figure S9**: Product ion mass spectrum of KP-54 M7, *m*/*z* 1120 (z = 3), NCE: 30%. 5](#_Toc224658921)

[**Figure S10**: Product ion mass spectrum of KP-54 M8, *m*/*z* 1114 (z = 3), NCE: 30%. 5](#_Toc224658922)

[**Figure S11**: Product ion mass spectrum of KP-54 M9, *m*/*z* 871 (z = 2), NCE: 30%. 5](#_Toc224658923)

[**Figure S12**: Product ion mass spectrum of KP-54 M10, *m*/*z* 1519 (z = 2), NCE: 30%. 5](#_Toc224658924)

[**Figure S13**: Product ion mass spectrum of KP-14 M1, *m*/*z* 724 (z = 1), NCE: 30%. 5](#_Toc224658925)

[**Figure S14**: Product ion mass spectrum of KP-14 M2, *m*/*z* 839 (z = 1), NCE: 30%. 5](#_Toc224658926)

[**Figure S15**: Product ion mass spectrum of KP-14 M3, *m*/*z* 725 (z = 1), NCE: 40%. 6](#_Toc224658927)

[**Figure S16**: Product ion mass spectrum of KP-14 M4, *m*/*z* 840 (z = 1), NCE: 30%. 6](#_Toc224658928)

[**Figure S17**: Product ion mass spectrum of KP-14 M5, *m*/*z* 636 (z = 2), NCE: 30%. 6](#_Toc224658929)

[**Figure S18**: Product ion mass spectrum of KP-14 M6, *m*/*z* 741 (z = 2), NCE: 30%. 6](#_Toc224658930)

[**Figure S19**: Product ion mass spectrum of KP-14 M7, *m*/*z* 513 (z = 2), NCE: 30%. 6](#_Toc224658931)

[**Figure S20**: Product ion mass spectrum of KP-14 M8, *m*/*z* 798 (z = 2), NCE: 30%. 6](#_Toc224658932)

[**Figure S21**: Product ion mass spectrum of KP-14 M1, *m*/*z* 570 (z = 2), NCE: 30%. 7](#_Toc224658933)

[**Figure S22**: Product ion mass spectrum of KP-14 M10, *m*/*z* 814 (z = 2), NCE: 35%. 7](#_Toc224658934)

[**Figure S23**: Product ion mass spectrum of KP-13 M1, *m*/*z* 711 (z = 1), NCE: 30%. 7](#_Toc224658935)

[**Figure S24**: Product ion mass spectrum of KP-13 M2, *m*/*z* 724 (z = 1), NCE: 30%. 7](#_Toc224658936)

[**Figure S25**: Product ion mass spectrum of KP-13 M3, *m*/*z* 839 (z = 1), NCE: 30%. 7](#_Toc224658937)

[**Figure S26**: Product ion mass spectrum of KP-13 M4, *m*/*z* 725 (z = 1), NCE: 40%. 7](#_Toc224658938)

[**Figure S27**: Product ion mass spectrum of KP-13 M5, *m*/*z* 636 (z = 2), NCE: 30%. 8](#_Toc224658939)

[**Figure S28**: Product ion mass spectrum of KP-13 M6, *m*/*z* 945 (z = 1), NCE: 40%. 8](#_Toc224658940)

[**Figure S29**: Product ion mass spectrum of KP-13 M7, *m*/*z* 1026 (z = 1), NCE: 40%. 8](#_Toc224658941)

[**Figure S30**: Product ion mass spectrum of KP-13 M8, *m*/*z* 741 (z = 2), NCE: 30%. 8](#_Toc224658942)

[**Figure S31**: Product ion mass spectrum of KP-13 M9, *m*/*z* 570 (z = 2), NCE: 30%. 8](#_Toc224658943)

[**Figure S32**: Product ion mass spectrum of KP-13 M10, *m*/*z* 652 (z = 2), NCE: 30%. 8](#_Toc224658944)

[**Figure S33**: Product ion mass spectrum of KP-10 M1, *m*/*z* 752 (z = 1), NCE: 30%. 9](#_Toc224658945)

[**Figure S34**: Product ion mass spectrum of KP-10 M2, *m*/*z* 839 (z = 1), NCE: 30%. 9](#_Toc224658946)

[**Figure S35**: Product ion mass spectrum of KP-10 M3, *m*/*z* 725 (z = 1), NCE: 30%. 9](#_Toc224658947)

[**Figure S36**: Product ion mass spectrum of KP-10 M4, *m*/*z* 578 (z = 2), NCE: 30%. 9](#_Toc224658948)

[**Figure S37**: Product ion mass spectrum of KP-10 M5, *m*/*z* 840 (z = 1), NCE: 30%. 9](#_Toc224658949)

[**Figure S38**: Product ion mass spectrum of KP-10 M6, *m*/*z* 830 (z = 1), NCE: 30%. 9](#_Toc224658950)

[**Figure S39**: Product ion mass spectrum of KP-10 M7, *m*/*z* 570 (z = 2), NCE: 30%. 10](#_Toc224658951)

[**Figure S40**: Product ion mass spectrum of KP-10 M8, *m*/*z* 652 (z = 2), NCE: 30%. 10](#_Toc224658952)

[**Figure S41**: Product ion mass spectrum of KP-10 M9, *m*/*z* 653 (z = 2), NCE: 30%. 10](#_Toc224658953)

[**Figure S42**: Product ion mass spectrum of KP-10 M10, *m*/*z* 1000 (z = 1), NCE: 30%. 10](#_Toc224658954)

[**Figure S43**: Product ion mass spectrum of TAK-448 M1, *m*/*z* 430 (z = 1), NCE: 30%. 10](#_Toc224658955)

[**Figure S44**: Product ion mass spectrum of TAK-448 M2, *m*/*z* 545 (z = 1), NCE: 35%. 10](#_Toc224658956)

[**Figure S45**: Product ion mass spectrum of TAK-448 M3, *m*/*z* 692 (z = 1), NCE: 30%. 11](#_Toc224658957)

[**Figure S46**: Product ion mass spectrum of TAK-448 M4, *m*/*z* 793 (z = 1), NCE: 30%. 11](#_Toc224658958)

[**Figure S47**: Product ion mass spectrum of TAK-448 M5, *m*/*z* 699 (z = 1), NCE: 30%. 11](#_Toc224658959)

[**Figure S48**: Product ion mass spectrum of TAK-448 M6, *m*/*z* 1040 (z = 1), NCE: 30%. 11](#_Toc224658960)

[**Figure S49**: Product ion mass spectrum of TAK-448 M7, *m*/*z* 615 (z = 1), NCE: 25%. 11](#_Toc224658961)

[**Figure S50**: Product ion mass spectrum of TAK-448 M8, *m*/*z* 557 (z = 1), NCE: 30%. 11](#_Toc224658962)

[**Figure S51**: Product ion mass spectrum of TAK-448 M9, *m*/*z* 571 (z = 1), NCE: 30%. 12](#_Toc224658963)

[**Figure S52**: Product ion mass spectrum of TAK-448 M10, *m*/*z* 1227 (z = 1), NCE: 35%. 12](#_Toc224658964)

[**Figure S53**: Proposed alternative structure of TAK-448 M7. 12](#_Toc224658965)

**
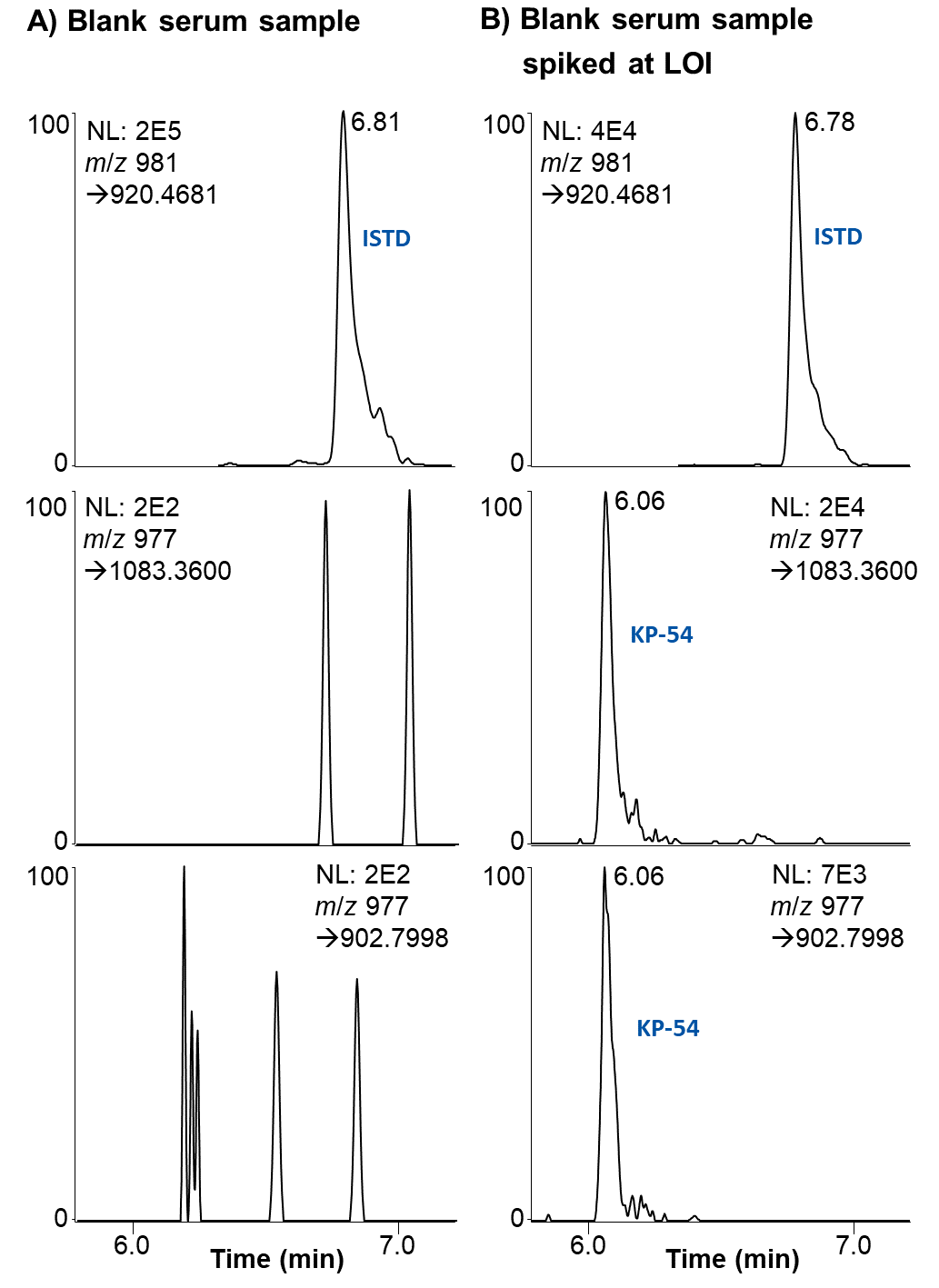
**

**Figure S1**: Representative extracted ion chromatograms of a blank serum sample (A) and a serum sample spiked with KP-54 at the limit of identification corresponding to 0.8 ng/ml (B).

**
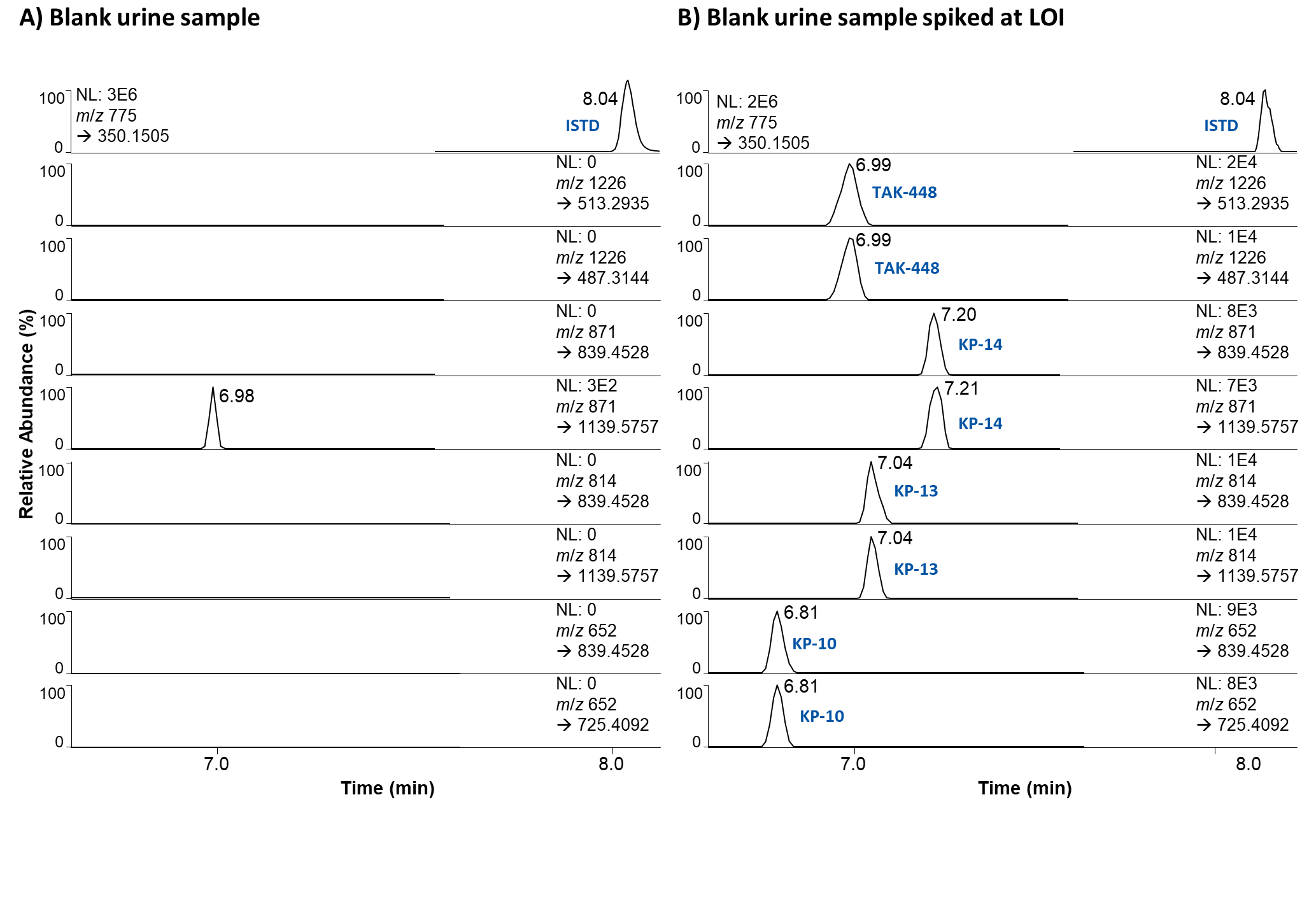
**

**Figure S2**: Representative extracted ion chromatograms of a blank urine sample (A) and a urine sample spiked at the limit of identification corresponding to 0.01 ng/ml (TAK-448), 0.2 ng/ml (KP-14), 0.1 ng/ml (KP-13), and 0.05 ng/ml (KP-10), respectively (B).

**
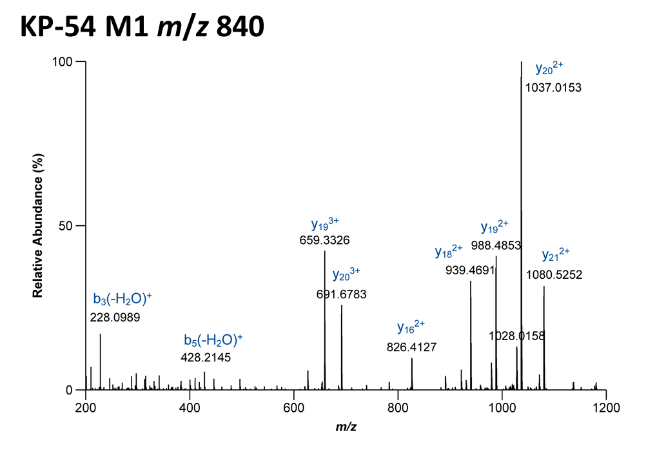
**

**Figure S3**: Product ion mass spectrum of KP-54 M1, *m*/*z* 840 (z = 3), NCE: 30%.

**
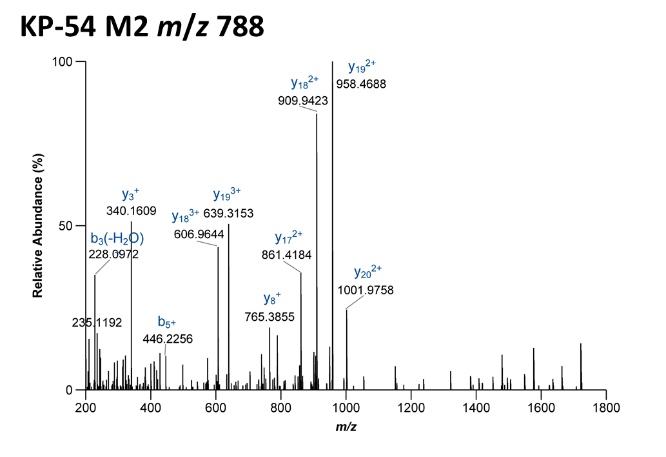
**

**Figure S4**: Product ion mass spectrum of KP-54 M2, *m*/*z* 788 (z = 3), NCE: 30%.

**
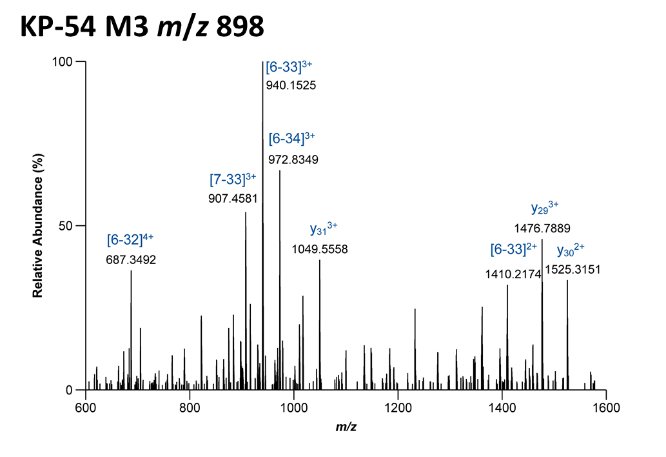
**

**Figure S5**: Product ion mass spectrum of KP-54 M3, *m*/*z* 898 (z = 4), NCE: 30%.

**
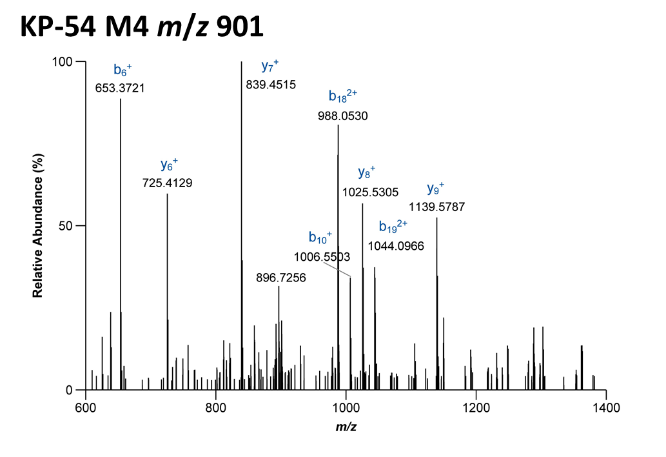
**

**Figure S6**: Product ion mass spectrum of KP-54 M4, *m*/*z* 901 (z = 4), NCE: 30%.

**
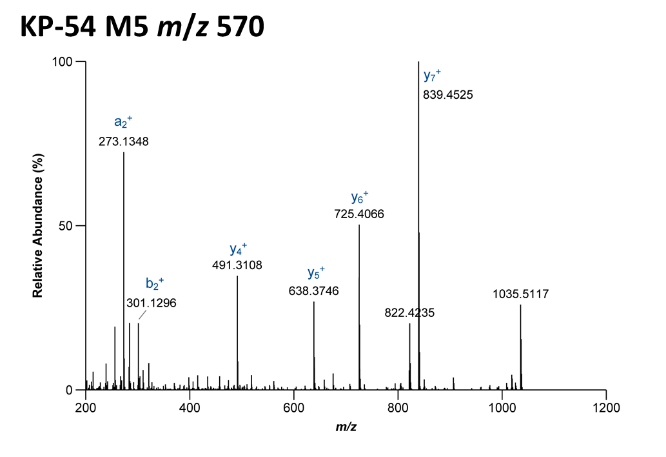
**

**Figure S7**: Product ion mass spectrum of KP-54 M5, *m*/*z* 570 (z = 2), NCE: 30%.

**
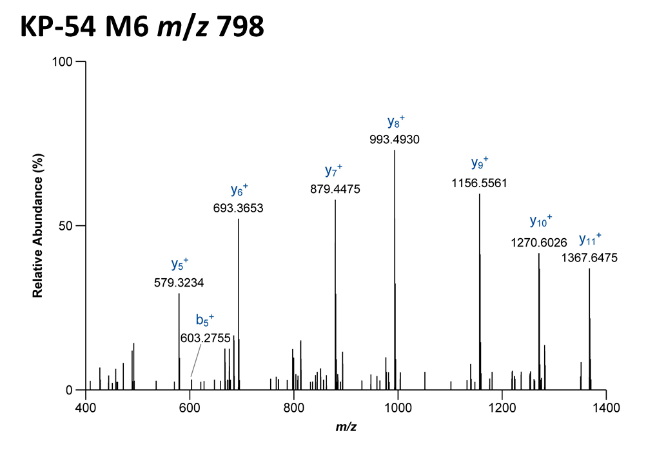
**

**Figure S8**: Product ion mass spectrum of KP-54 M6, *m*/*z* 798 (z = 2), NCE: 30%.

**
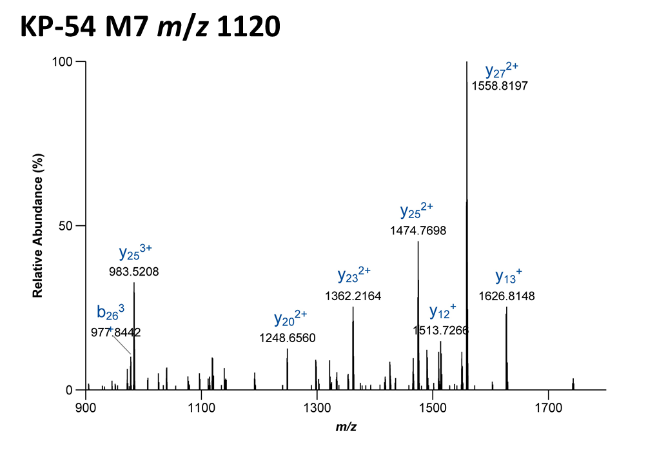
**

**Figure S9**: Product ion mass spectrum of KP-54 M7, *m*/*z* 1120 (z = 3), NCE: 30%.

**
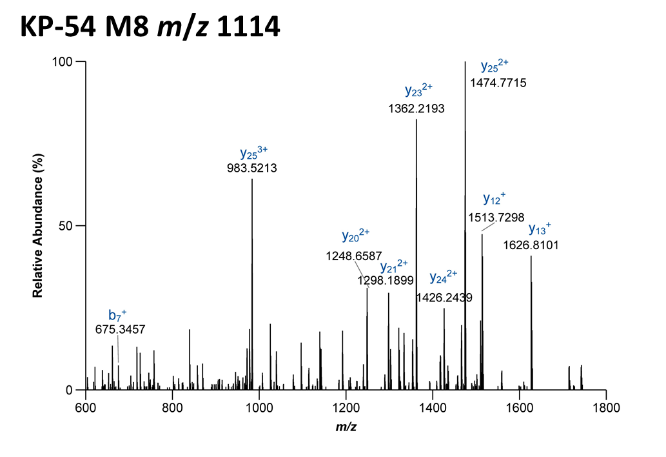
**

**Figure S10**: Product ion mass spectrum of KP-54 M8, *m*/*z* 1114 (z = 3), NCE: 30%.

**
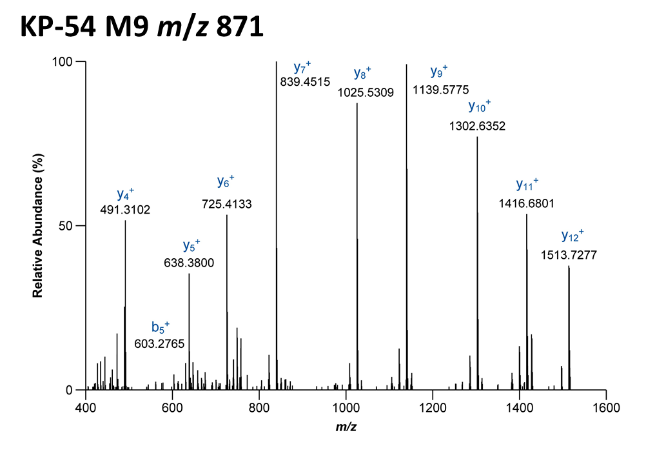
**

**Figure S11**: Product ion mass spectrum of KP-54 M9, *m*/*z* 871 (z = 2), NCE: 30%.

**
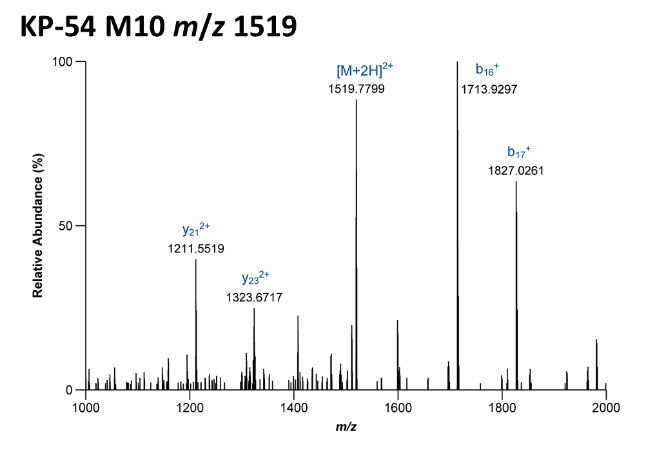
**

**Figure S12**: Product ion mass spectrum of KP-54 M10, *m*/*z* 1519 (z = 2), NCE: 30%.

**
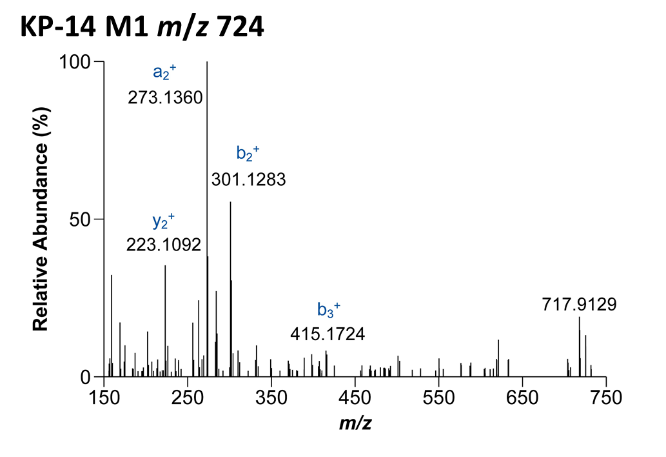
**

**Figure S13**: Product ion mass spectrum of KP-14 M1, *m*/*z* 724 (z = 1), NCE: 30%.

**
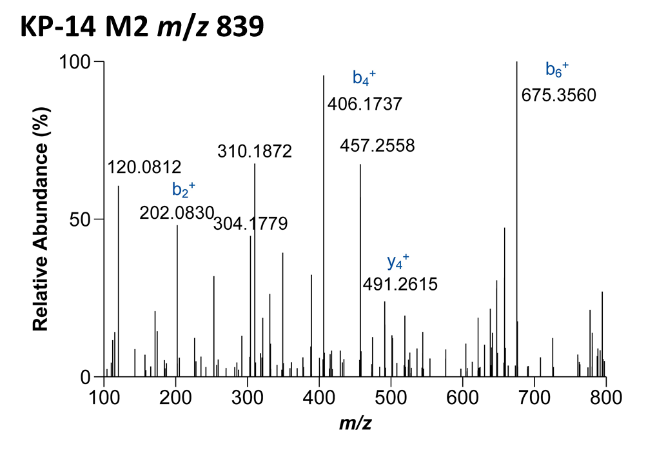
**

**Figure S14**: Product ion mass spectrum of KP-14 M2, *m*/*z* 839 (z = 1), NCE: 30%.

**
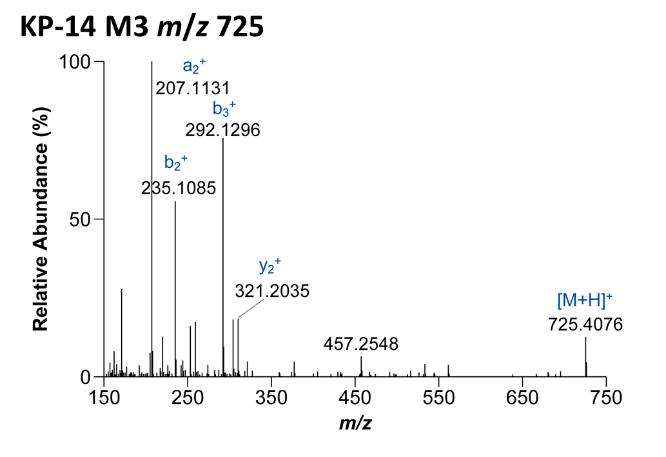
**

**Figure S15**: Product ion mass spectrum of KP-14 M3, *m*/*z* 725 (z = 1), NCE: 40%.

**
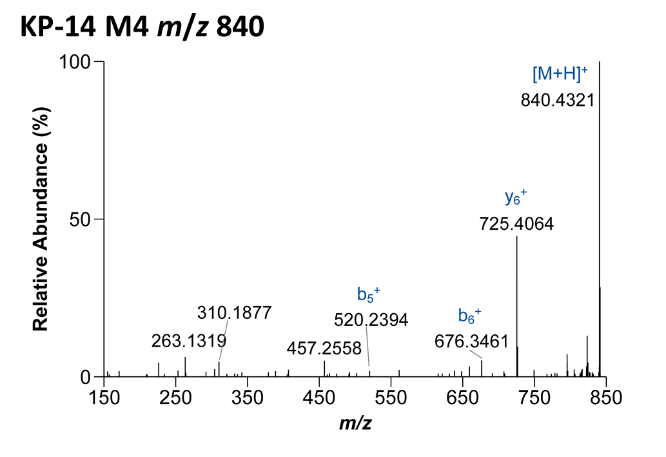
**

**Figure S16**: Product ion mass spectrum of KP-14 M4, *m*/*z* 840 (z = 1), NCE: 30%.

**
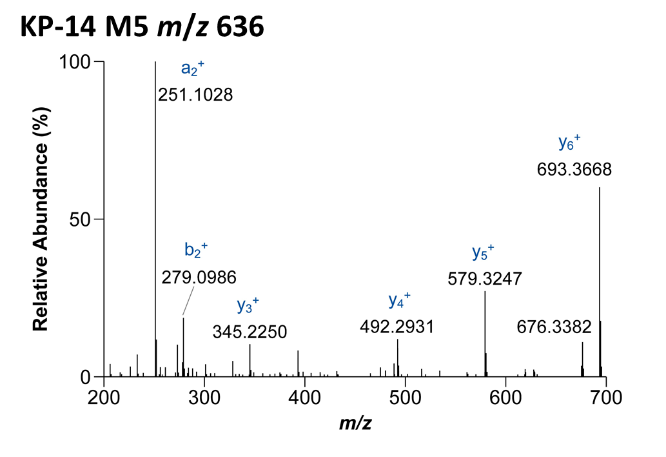
**

**Figure S17**: Product ion mass spectrum of KP-14 M5, *m*/*z* 636 (z = 2), NCE: 30%.

**
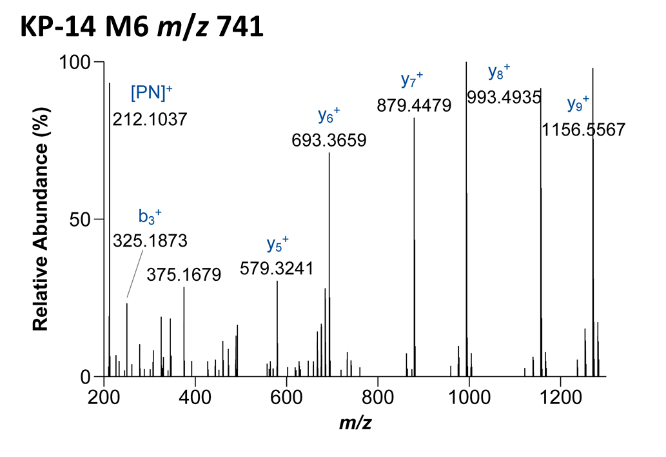
**

**Figure S18**: Product ion mass spectrum of KP-14 M6, *m*/*z* 741 (z = 2), NCE: 30%.

**
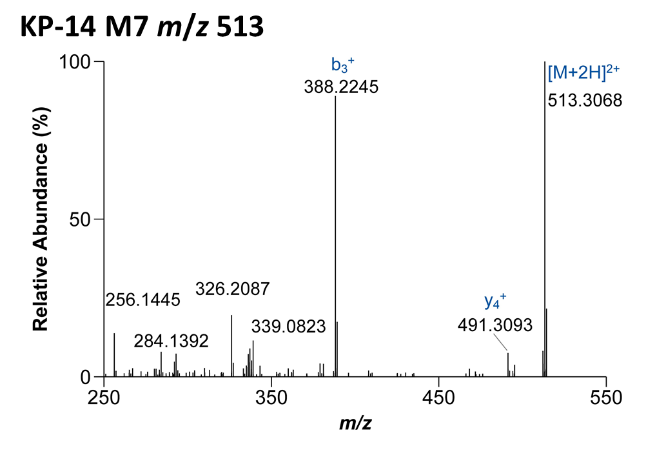
**

**Figure S19**: Product ion mass spectrum of KP-14 M7, *m*/*z* 513 (z = 2), NCE: 30%.

**
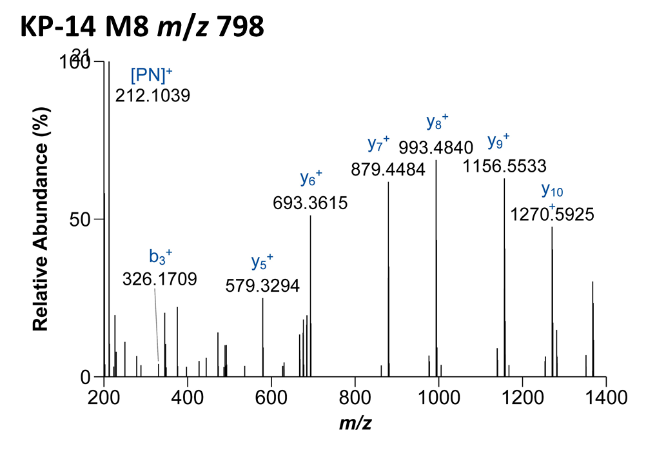
**

**Figure S20**: Product ion mass spectrum of KP-14 M8, *m*/*z* 798 (z = 2), NCE: 30%.

**
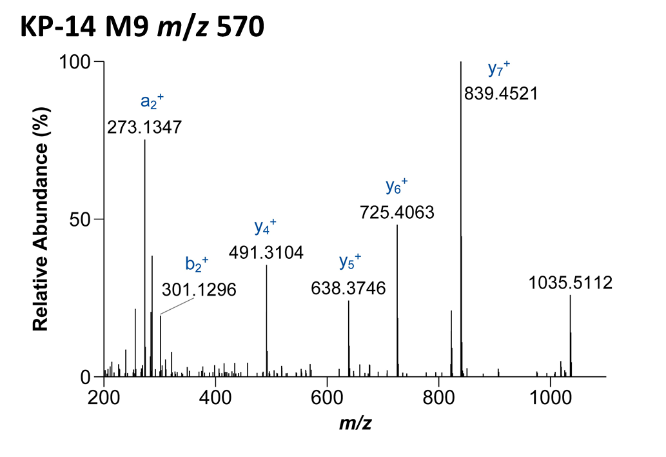
**

**Figure S21**: Product ion mass spectrum of KP-14 M1, *m*/*z* 570 (z = 2), NCE: 30%.

**
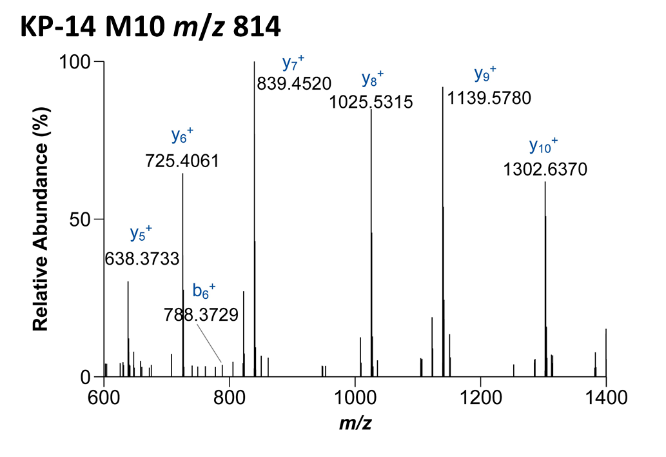
**

**Figure S22**: Product ion mass spectrum of KP-14 M10, *m*/*z* 814 (z = 2), NCE: 35%.

**
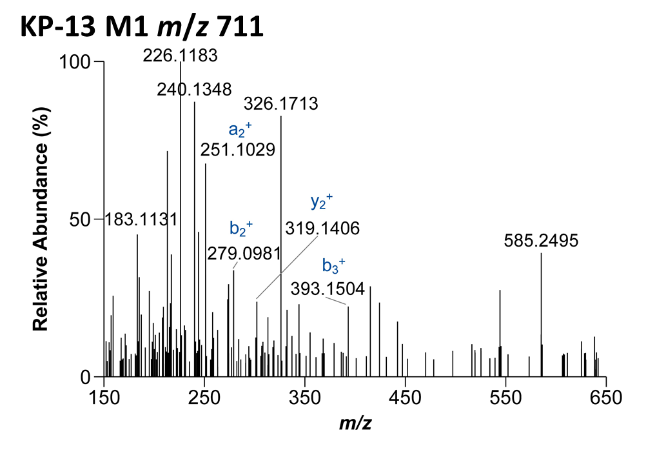
**

**Figure S23**: Product ion mass spectrum of KP-13 M1, *m*/*z* 711 (z = 1), NCE: 30%.

**
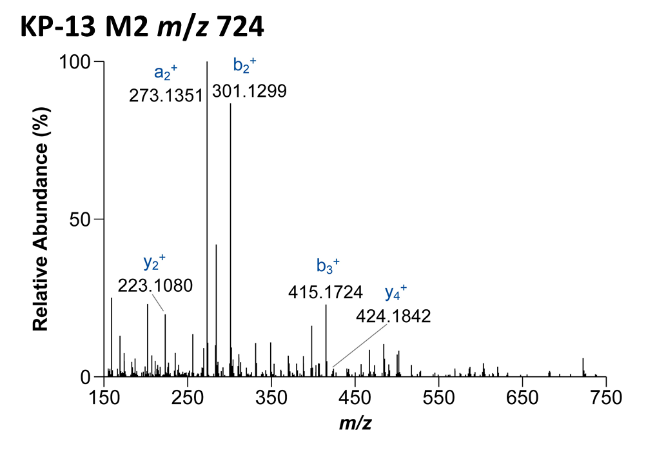
**

**Figure S24**: Product ion mass spectrum of KP-13 M2, *m*/*z* 724 (z = 1), NCE: 30%.

**
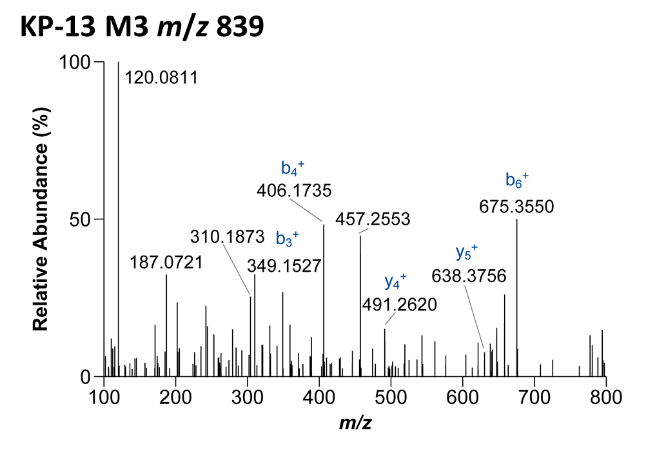
**

**Figure S25**: Product ion mass spectrum of KP-13 M3, *m*/*z* 839 (z = 1), NCE: 30%.

**
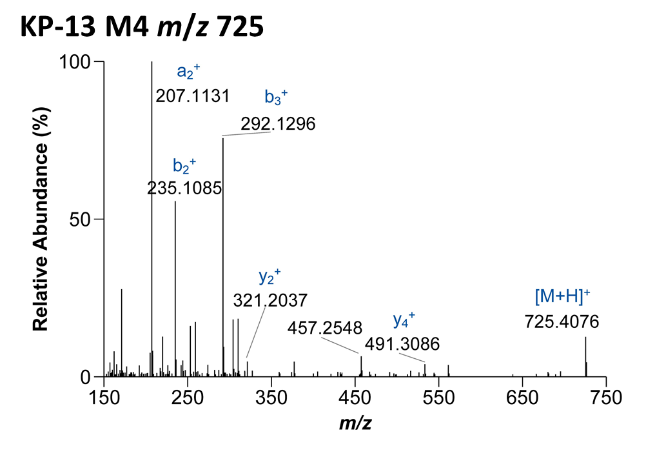
**

**Figure S26**: Product ion mass spectrum of KP-13 M4, *m*/*z* 725 (z = 1), NCE: 40%.

**
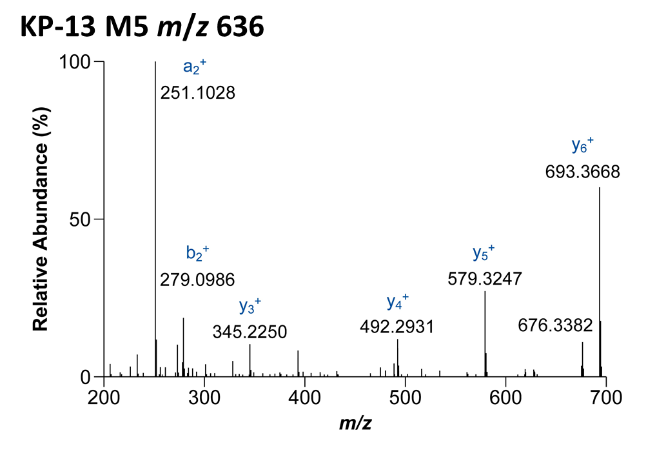
**

**Figure S27**: Product ion mass spectrum of KP-13 M5, *m*/*z* 636 (z = 2), NCE: 30%.

**
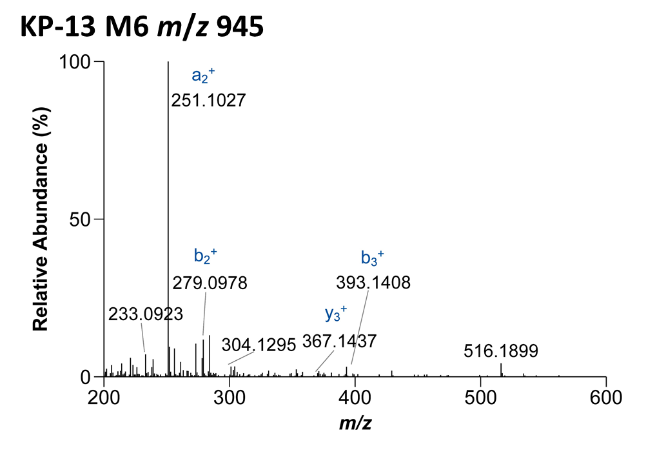
**

**Figure S28**: Product ion mass spectrum of KP-13 M6, *m*/*z* 945 (z = 1), NCE: 40%.

**
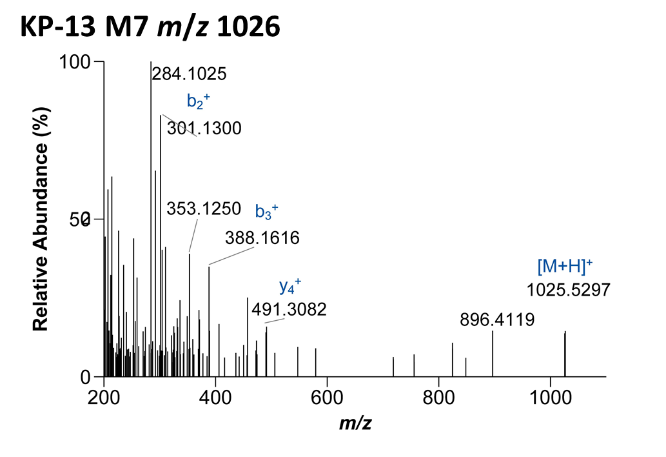
**

**Figure S29**: Product ion mass spectrum of KP-13 M7, *m*/*z* 1026 (z = 1), NCE: 40%.

**
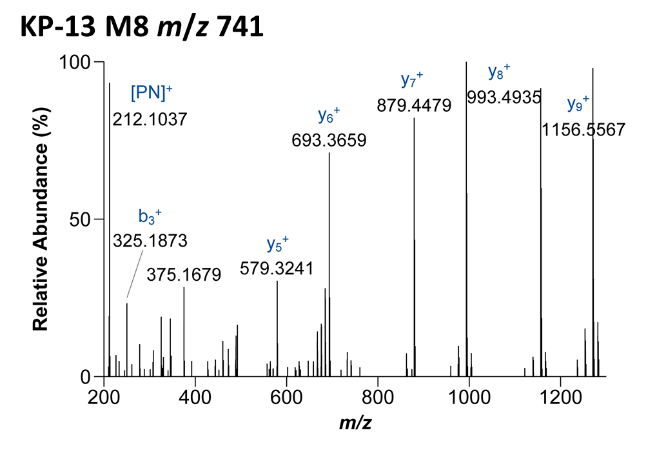
**

**Figure S30**: Product ion mass spectrum of KP-13 M8, *m*/*z* 741 (z = 2), NCE: 30%.

**
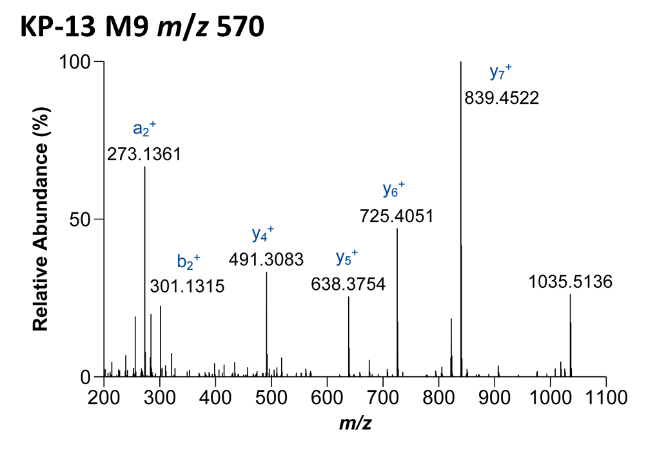
**

**Figure S31**: Product ion mass spectrum of KP-13 M9, *m*/*z* 570 (z = 2), NCE: 30%.

**
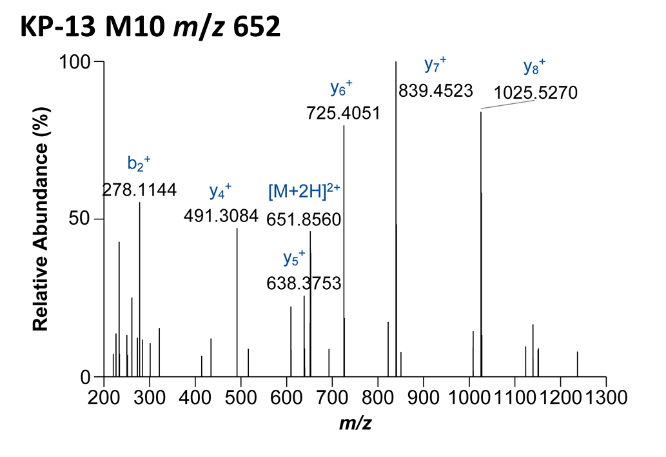
**

**Figure S32**: Product ion mass spectrum of KP-13 M10, *m*/*z* 652 (z = 2), NCE: 30%.

**
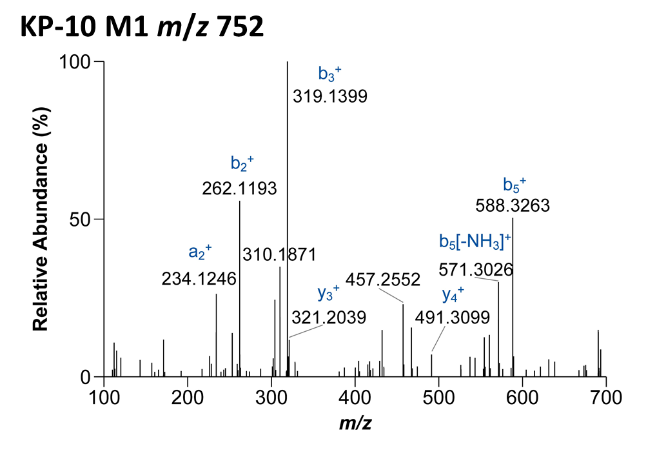
**

**Figure S33**: Product ion mass spectrum of KP-10 M1, *m*/*z* 752 (z = 1), NCE: 30%.

**
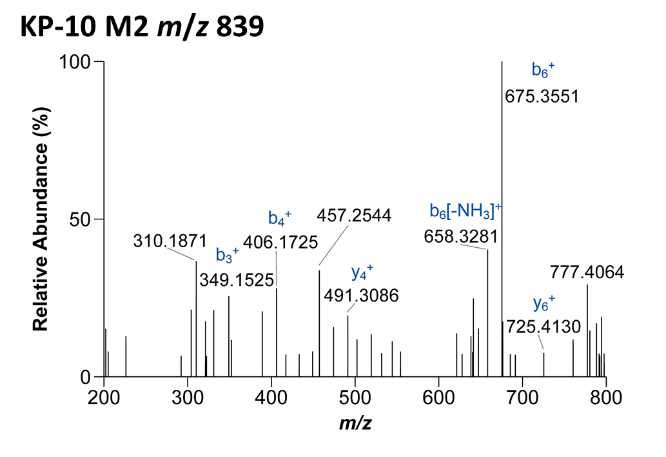
**

**Figure S34**: Product ion mass spectrum of KP-10 M2, *m*/*z* 839 (z = 1), NCE: 30%.

**
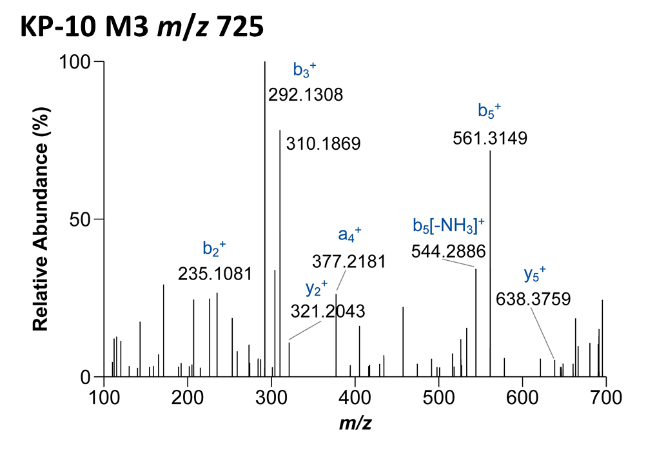
**

**Figure S35**: Product ion mass spectrum of KP-10 M3, *m*/*z* 725 (z = 1), NCE: 30%.

**
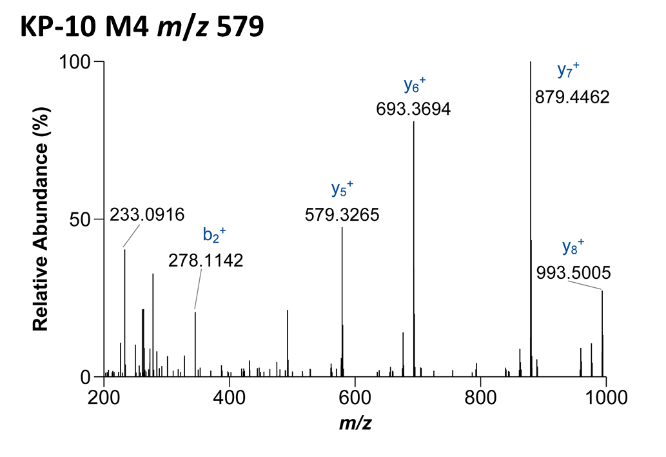
**

**Figure S36**: Product ion mass spectrum of KP-10 M4, *m*/*z* 578 (z = 2), NCE: 30%.

**
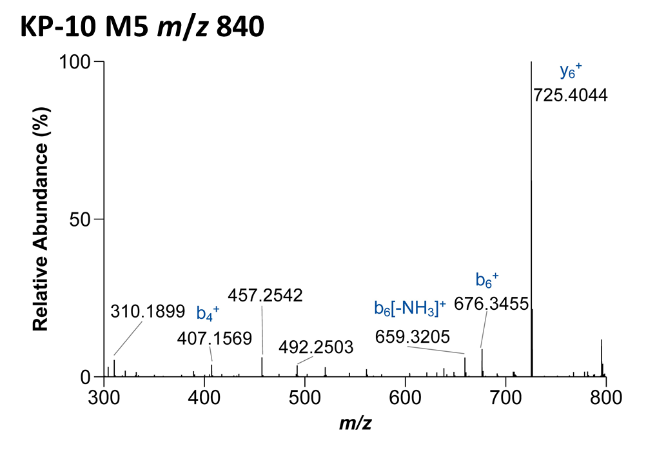
**

**Figure S37**: Product ion mass spectrum of KP-10 M5, *m*/*z* 840 (z = 1), NCE: 30%.

**
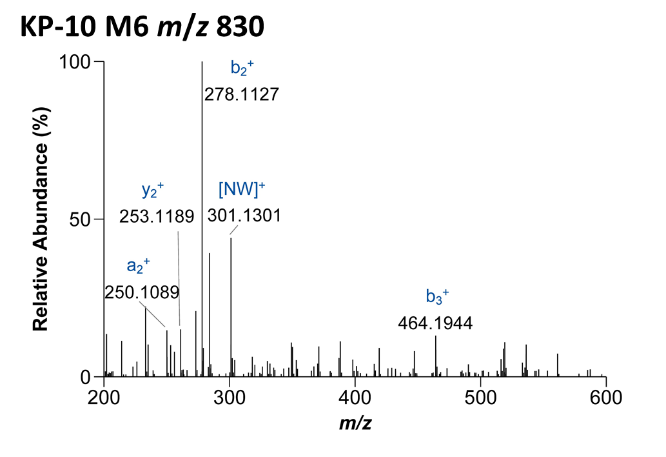
**

**Figure S38**: Product ion mass spectrum of KP-10 M6, *m*/*z* 830 (z = 1), NCE: 30%.

**
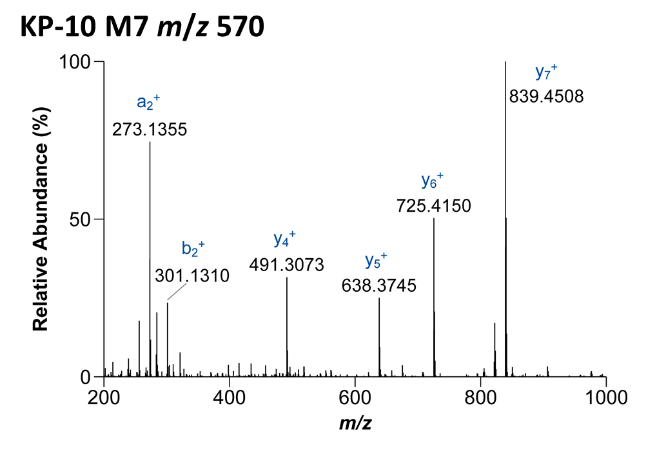
**

**Figure S39**: Product ion mass spectrum of KP-10 M7, *m*/*z* 570 (z = 2), NCE: 30%.

**
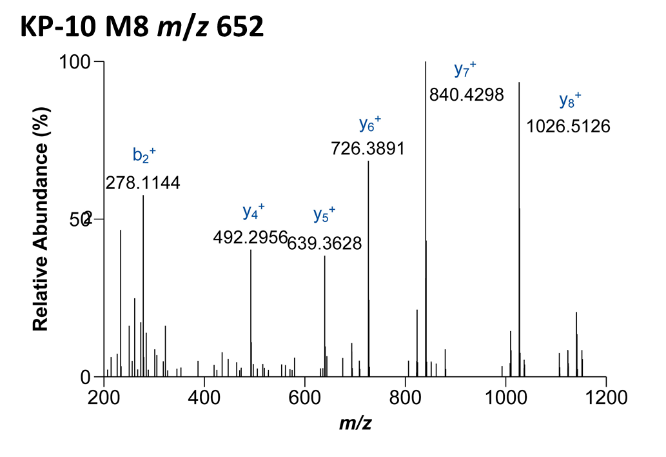
**

**Figure S40**: Product ion mass spectrum of KP-10 M8, *m*/*z* 652 (z = 2), NCE: 30%.

**
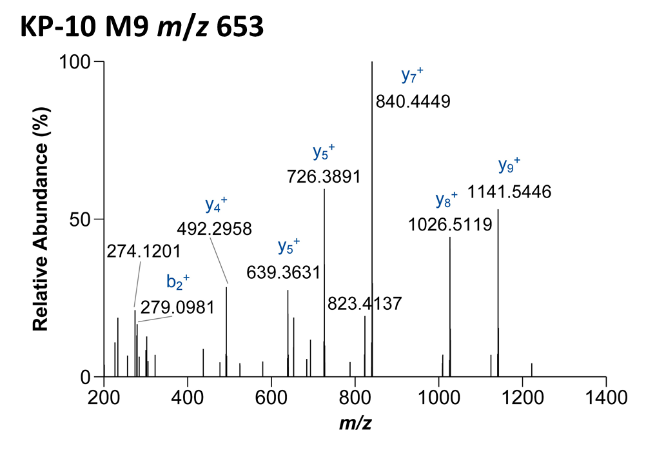
**

**Figure S41**: Product ion mass spectrum of KP-10 M9, *m*/*z* 653 (z = 2), NCE: 30%.

**
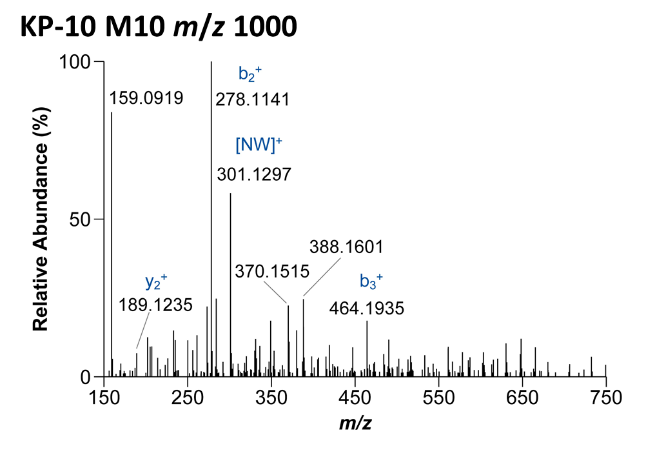
**

**Figure S42**: Product ion mass spectrum of KP-10 M10, *m*/*z* 1000 (z = 1), NCE: 30%.

**
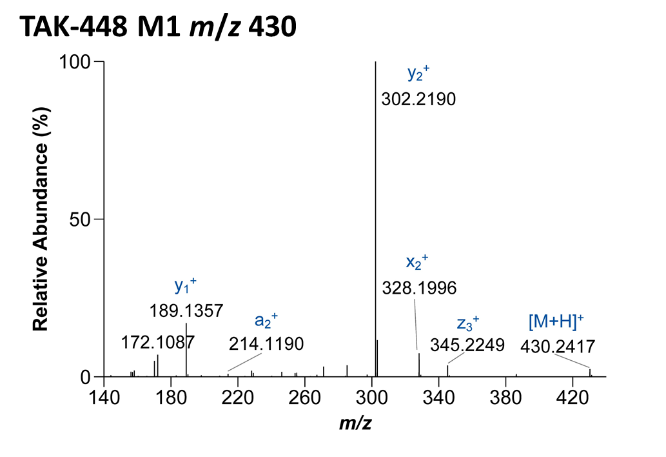
**

**Figure S43**: Product ion mass spectrum of TAK-448 M1, *m*/*z* 430 (z = 1), NCE: 30%.

**
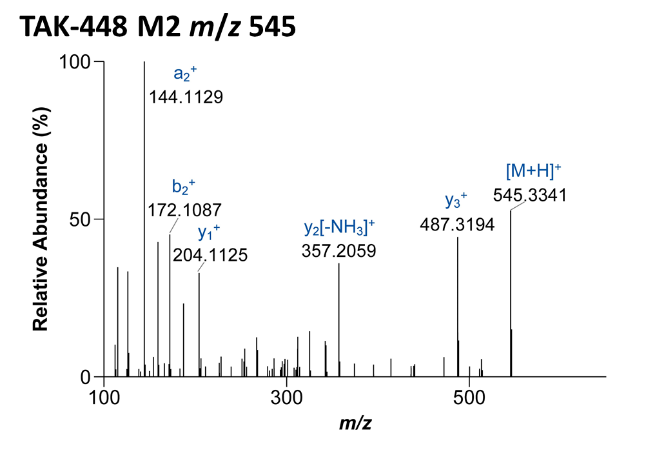
**

**Figure S44**: Product ion mass spectrum of TAK-448 M2, *m*/*z* 545 (z = 1), NCE: 35%.

**
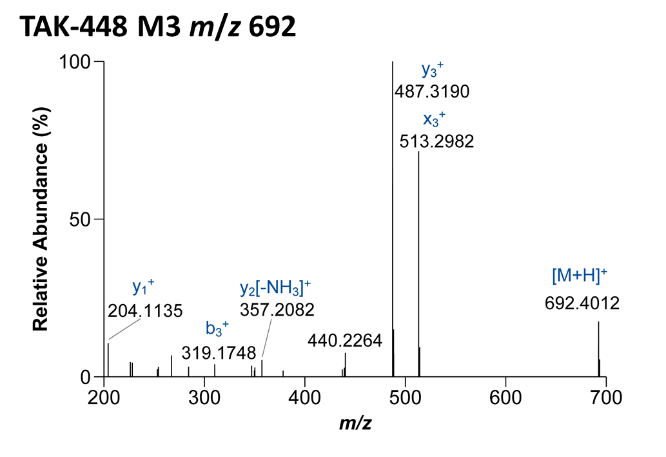
**

**Figure S45**: Product ion mass spectrum of TAK-448 M3, *m*/*z* 692 (z = 1), NCE: 30%.

**
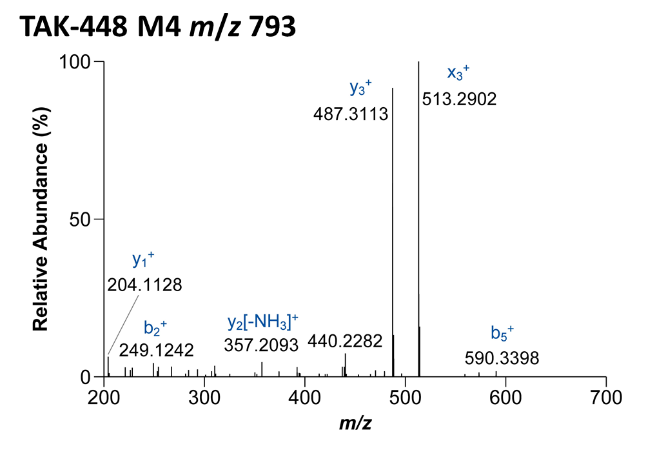
**

**Figure S46**: Product ion mass spectrum of TAK-448 M4, *m*/*z* 793 (z = 1), NCE: 30%.

**
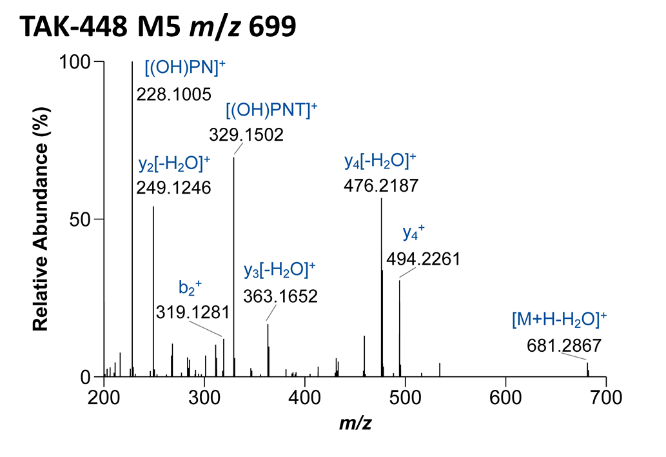
**

**Figure S47**: Product ion mass spectrum of TAK-448 M5, *m*/*z* 699 (z = 1), NCE: 30%.

**
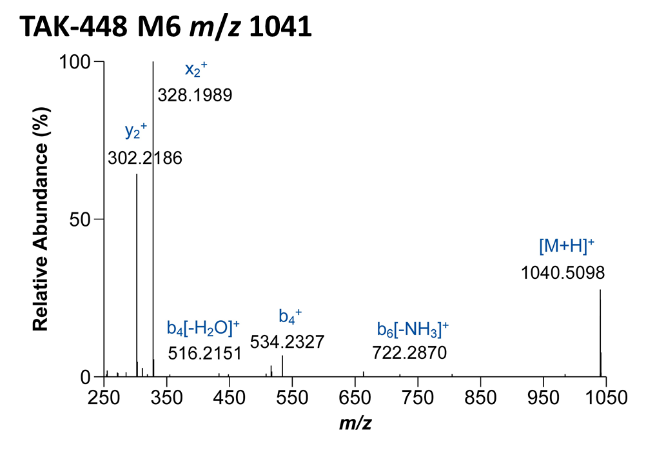
**

**Figure S48**: Product ion mass spectrum of TAK-448 M6, *m*/*z* 1040 (z = 1), NCE: 30%.

**
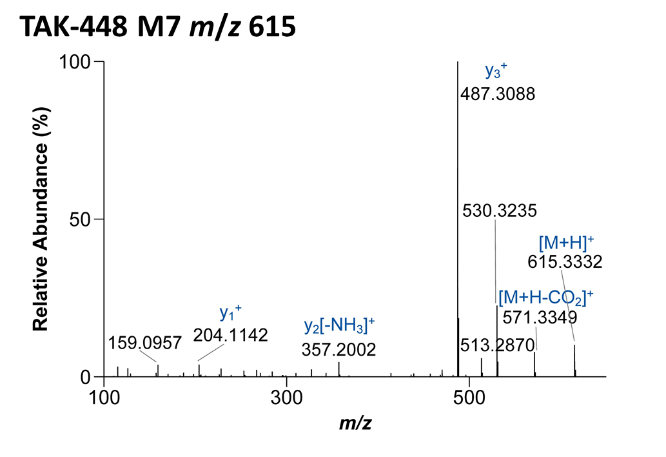
**

**Figure S49**: Product ion mass spectrum of TAK-448 M7, *m*/*z* 615 (z = 1), NCE: 25%.

**
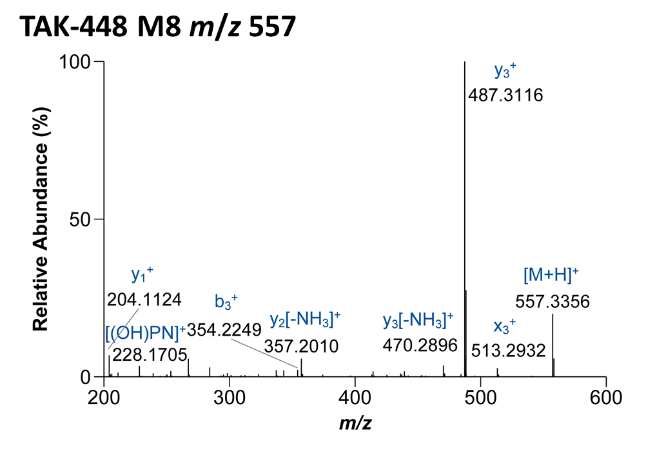
**

**Figure S50**: Product ion mass spectrum of TAK-448 M8, *m*/*z* 557 (z = 1), NCE: 30%.

**
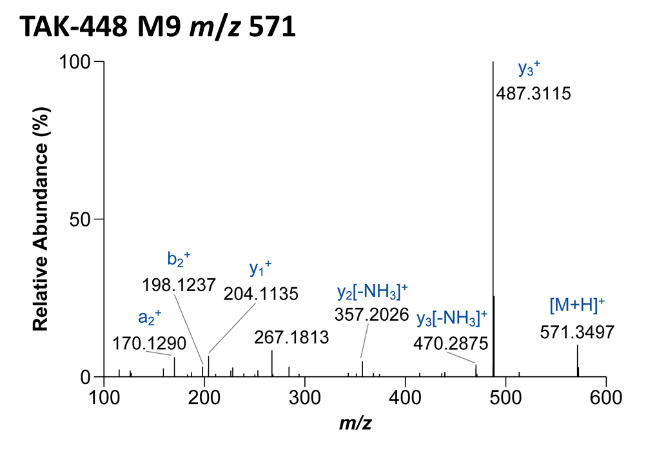
**

**Figure S51**: Product ion mass spectrum of TAK-448 M9, *m*/*z* 571 (z = 1), NCE: 30%.

**
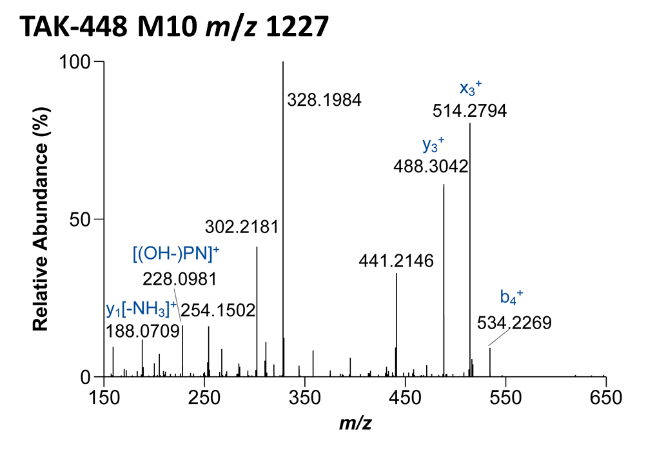
**

**Figure S52**: Product ion mass spectrum of TAK-448 M10, *m*/*z* 1227 (z = 1), NCE: 35%.

**
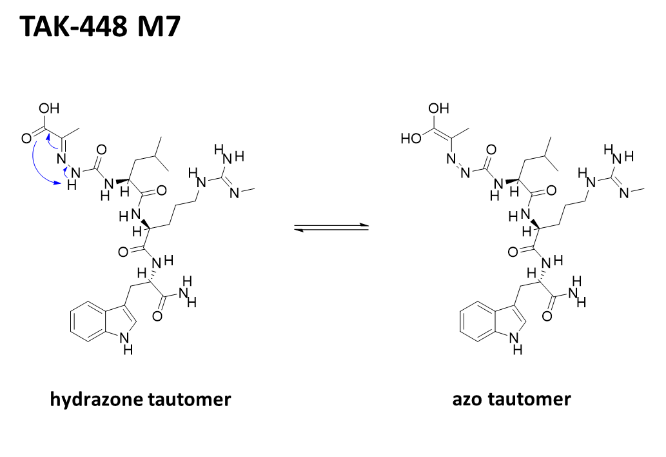
**

**Figure S53**: Proposed alternative structure of TAK-448 M7.
